# Transcriptional and translational dynamics underlying heat shock response in the thermophilic crenarchaeon *Sulfolobus acidocaldarius*

**DOI:** 10.1128/mbio.03593-22

**Published:** 2023-08-29

**Authors:** Rani Baes, Felix Grünberger, Sébastien Pyr dit Ruys, Mohea Couturier, Sarah De Keulenaer, Sonja Skevin, Filip Van Nieuwerburgh, Didier Vertommen, Dina Grohmann, Sébastien Ferreira-Cerca, Eveline Peeters

**Affiliations:** 1 Research Group of Microbiology, Department of Bioengineering Sciences, Vrije Universiteit Brussel, Brussels, Belgium; 2 Institute of Microbiology and Archaea Centre, Universität Regensburg, Regensburg, Germany; 3 Institut de Duve, Université Catholique de Louvain, Brussels, Belgium; 4 NXTGNT, Faculty of Pharmaceutical Sciences, Ghent University, Ghent, Belgium; 5 Cellular Biochemistry of Microorganisms, Biochemie III, Universität Regensburg, Regensburg, Germany; 6 Laboratoire de Biologie Structurale de la Cellule (BIOC), UMR 7654 -CNRS, Ecole polytechnique, Institut Polytechnique de Paris, Palaiseau, France; University of Würzburg, Würzburg, Germany; Christian-Albrechts-Universitat zu Kiel, Kiel, Germany

**Keywords:** archaea, *Sulfolobus*, heat shock, integrated omics, gene regulation

## Abstract

High-temperature stress is critical for all organisms and induces a profound cellular response. For Crenarchaeota, little information is available on how heat shock affects cellular processes and on how this response is regulated. We set out to study heat shock response in the thermoacidophilic model crenarchaeon *Sulfolobus acidocaldarius,* which thrives in volcanic hot springs and has an optimal growth temperature of 75°C. Pulse-labeling experiments demonstrated that a temperature shift to 86°C induces a drastic reduction of the transcriptional and translational activity, but that RNA and protein neosynthesis still occurs. By combining RNA sequencing and mass spectrometry, an integrated mapping of the transcriptome and proteome was performed. This revealed that heat shock causes an immediate change in the gene expression profile, with RNA levels of half of the genes being affected, followed by a more subtle reprogramming of the protein landscape. Functional enrichment analysis indicated that nearly all cellular processes are affected by heat shock. A limited correlation was observed in the differential expression on the RNA and protein level, suggesting a prevalence of post-transcriptional and post-translational regulation. Furthermore, promoter sequence analysis of heat shock regulon genes demonstrated the conservation of strong transcription initiation elements for highly induced genes, but an absence of a conserved protein-binding motif. It is, therefore, hypothesized that histone-lacking archaea such as Sulfolobales use an evolutionarily ancient regulatory mechanism that relies on temperature-responsive changes in DNA organization and compaction induced by the action of nucleoid-associated proteins, as well as on enhanced recruitment of initiation factors.

**IMPORTANCE:**

Heat shock response is the ability to respond adequately to sudden temperature increases that could be harmful for cellular survival and fitness. It is crucial for microorganisms living in volcanic hot springs that are characterized by high temperatures and large temperature fluctuations. In this study, we investigated how *S. acidocaldarius*, which grows optimally at 75°C, responds to heat shock by altering its gene expression and protein production processes. We shed light on which cellular processes are affected by heat shock and propose a hypothesis on underlying regulatory mechanisms. This work is not only relevant for the organism’s lifestyle, but also with regard to its evolutionary status. Indeed, *S. acidocaldarius* belongs to the archaea, an ancient group of microbes that is more closely related to eukaryotes than to bacteria. Our study thus also contributes to a better understanding of the early evolution of heat shock response.

## INTRODUCTION

Crenarchaeota belonging to the Sulfolobales are characterized by a thermoacidophilic lifestyle with an optimal growth temperature between 75°C and 80°C, enabling them to thrive in geothermal hot springs, solfataras, or mud pools (1). Such high-temperature habitats are typically characterized by large spatiotemporal temperature variations (2 – 5). These are caused by changes in flow rates, in the volcanic activity of the underlying hydrothermal aquifers, in seismic activity coupled to ground deformation and meteoric precipitation. It has been observed that large temperature fluctuations prevail along drainways and that daily temperature variations up to 10°C are common at a given location in the outflow channel (3). It can thus be anticipated that response to temperature stress, especially to heat shock, which compromises cellular integrity, makes up an inherent and important part of the stress response physiology of Sulfolobales. A sudden increase in temperature above the already high optimal growth temperature could lead to detrimental cellular damage, such as increased membrane permeability, damage of nucleic acids, or protein denaturation and aggregation (6). To counteract these effects, a heat shock response is initiated, consisting of a combination of universally conserved and (cren-)archaeal-unique features (7).

In Sulfolobales, heat shock response leads to an altered lipid composition of the cytoplasmic membrane (8, 9) and an increase in positive supercoiling of the DNA, mediated by an increased activity of reverse gyrase TopR1 at elevated temperatures (10 – 12). In addition, heat shock triggers the upregulation of heat shock proteins (HSPs), which are universal key players in maintaining protein homeostasis by functioning as molecular chaperones (6, 7). Like in other archaea, and in contrast to eukaryotes and bacteria, the HSP machinery of Sulfolobales consists of a limited set of components (7). Small HSPs (sHSPs) and prefoldin bind to denaturing proteins, thereby protecting them from aggregation, and shuttle them to the HSP60-type group II chaperonin complex, also referred to as the thermosome (13 – 16). The latter forms a large complex that refolds denatured proteins in an ATP-dependent manner. Sulfolobales encode three different thermosome subunits (Thα, Thβ, and Thγ), with each subunit being differentially expressed in response to temperature changes, leading to thermosome complexes with different subunit compositions and substrate specificities (17, 18). At elevated temperatures, the thermosome complex of *Saccharolobus solfataricus* shifts from an octameric composition with alternating Thα and Thβ subunits at physiological temperature to an all-Thβ non-americ composition in *in vitro* conditions (18).

Given the far-reaching physiological effects of heat shock, it can be anticipated that an extensive gene regulatory program is induced, which is most likely not limited to the differential expression of HSP subunits. Indeed, a transcriptomic analysis has revealed that about one-third of all transcripts in *Sa. solfataricus* displays a differential abundance at 5 min after reaching a stable heat shock temperature (19). Also, *vapBC*-like toxin-antitoxin systems have been shown to be differentially expressed and to play a role in heat shock response (19 – 21). The extensive heat shock responsive transcriptional regulation was shown to be a transient mechanism, with most transcript levels returning to their baseline levels after prolonged heat shock (19). Moreover, also on a protein level regulation can be observed. Recently, a quantitative proteomics study was performed in the related species *Sulfolobus islandicus*, comparing growth at physiological and elevated temperatures (75°C and 85°C, respectively), demonstrating altered abundance levels of a wide range of proteins belonging to different functional categories (22).

Regulatory mechanisms that underly heat shock response are well understood in eukaryotic and bacterial systems, in which regulation is mostly dependent on global transcriptional regulators, such as the eukaryotic HSF1 activator or alternative σ factors in bacteria (e.g., σ^32^ in *Escherichia coli*) (6). In archaea, much less is known about heat shock responsive gene regulation. The only exception is that certain Euryarchaeota have been shown to harbor a dedicated transcription factor that exerts regulation in response to heat shock: this factor is named Phr in *Pyrococcus furiosus* (23 – 25) and HSR1 in *Archaeoglobus fulgidus* (26). Phr and HSR1 have a similar regulon, including an sHSP-encoding gene and a gene encoding an AAA^+^ ATPase involved in heat shock response. However, their regulons do not include genes encoding key HSPs such as the thermosome subunits, suggesting that other regulatory mechanisms might be involved in establishing a complex regulatory network (7).

As opposed to Euryarchaeota, the regulatory mechanisms underlying heat shock responsive transcriptomic and proteomic dynamics are elusive in Crenarchaeota and more specifically, in Sulfolobales. Moreover, while previous studies have revealed large alterations in transcript and protein levels in response to high-temperature stress (19, 22), it is unknown whether these changes are mainly resulting from transcriptional, post-transcriptional, and/or post-translational regulation or also from proteolytic and/or RNA degradation effects. In this study, we shed light on heat shock responsive regulation in the model crenarchaeal species *Sulfolobus acidocaldarius* by performing an integrated mapping of the transcriptomic and proteomic landscape. To this end, we combine RNA and protein pulse-labeling, RNA sequencing (RNA-seq), and mass spectrometry (MS) methodologies. While improving the understanding of which key cellular processes are subjected to regulation, we also reveal how *de novo* transcription and translation are affected by heat shock and at which information processing levels regulation mainly takes place.

## MATERIALS AND METHODS

### Strains, growth conditions, and sample preparations

Starting from *S. acidocaldarius* strain SK-1 (27), genomic epitope tagging was performed to generate a strain expressing a FLAG-tagged thermosome α subunit, a 6xHis-tagged thermosome β subunit, and a human influenza hemagglutinin (HA)-tagged thermosome γ subunit (SK-1 × Thα-FLAG + Thβ-6xHis + Thγ-HA) (Supplementary Methods). All *S. acidocaldarius* strains used in this work (Table S1) were cultured in Brock basal salts medium (1) supplemented with 0.2% sucrose, 0.1% NZ-amine, and 20 µg mL^−1^ uracil, unless stated otherwise. For RNA pulse-labeling experiments, the same medium was used but supplemented with 5 µg mL^−1^ uracil. For protein pulse-labeling experiments, Brock basal salts medium was supplemented with 0.2% sucrose, 10 µg mL^−1^ uracil, and 0.73 g L^−1^ yeast methionine/uracil drop-out medium (CSM-Met-Ura, MP Biomedicals, USA, Solon) and lacked NZ-amine. All culture media were acidified to pH 3.0–3.5 with H_2_SO_4_. Cultures were grown at 75°C while shaking, unless stated otherwise, and growth was followed by measuring the optical density at 600 nm (OD_600nm_).

The experimental heat shock setup for the omic experiments was adapted from reference (28) (Fig. S1a). Briefly, four biological replicates of *S. acidocaldarius* MW001 cultures were grown until mid-exponential phase (OD_600nm_ 0.45) after which they were transferred to a six-well plate in a 75°C preheated shaking heating block. The temperature of the heating block was increased to trigger a heat shock at 86°C, and the samples were collected just before and at different time points during the heat shock. Each sample was split for transcriptomic and proteomic analysis. Samples for transcriptomic analysis were stabilized with an equal volume of RNAprotect Bacteria Reagent (Qiagen, USA, Maryland) before centrifugation (10 min at 6,574 × *g* and 4°C). Samples for proteomic analysis were additionally washed with 0.9% NaCl. The SK-1 × Thα-FLAG + Thβ−6xHis + Thγ-HA strain was included in the experimental setup to validate the induction of a heat shock response. Detailed information about the samples is presented in Data set S1. Cellular viability was confirmed in heat shock conditions by performing spot tests, and heat shock induction was assessed by western blotting (Supplementary Methods).

### RNA-seq and data analysis

RNA-seq analysis was performed by subjecting stabilized cell pellets to RNA extraction, followed by sample processing and high-throughput sequencing using an Illumina platform (Supplementary Methods). Data were processed by performing a quality control, mapping, and differential expression analysis by EdgeR (29) (Supplementary Methods, Data set S1 and S2).

### TMT-labeled liquid chromatography-tandem mass spectrometry and data analysis

Cell pellets were subjected to protein extraction, followed by further processing, tandem mass tag (TMT) labeling and a liquid chromatography-tandem mass spectrometry (LC-MS/MS) analysis (Supplementary Methods). The resulting MS/MS data were further processed, and differential expression was analyzed by DEqMS (30) as described (Supplementary Methods and Data set S2).

### arCOG analysis, correlation analysis of RNA-seq and MS data, and promoter analysis

Archaeal clusters of orthologous genes (arCOGs) classification was retrieved from reference (31), followed by manual curation. Gene set enrichment analysis of arCOGs was performed using the goseq package in R, which accounts for gene length bias (32). Next, *P*-values for over- and underrepresentation of arCOG terms in the differentially expressed genes were calculated separately for up- and downregulated genes based on RNA-seq and MS data, respectively, and were considered as either significantly enriched or not enriched below a cutoff of 0.05.

Pearson correlation coefficients were calculated from RNA-seq and MS log_2_ transformed count data and were used to analyze the association between the two methods.

Promoter motif analysis was performed by considering previously determined positions of primary transcription start sites (TSSs) (33). Position-specific sequences were extracted in a strand-specific way from −50 to +1 nt from each TSS and plotted in R using the ggseqlogo package (34). Complementary, the MEME command from the MEME Suite (35) was used for the discovery of ungapped motifs in upstream regions (-mod anr). Different combinations of MEME parameters (-pal, -revcomp) were tested with varying input lengths (−80 to +40, –50 to +1, and –120 to +1) and different regulation subsets to increase the sensitivity of motif discovery.

### Pulse labeling of neosynthesized RNA

RNA pulse-labeling experiments were performed for *S. acidocaldarius* SK-1 × Thα-FLAG + Thβ-6xHis + Thγ-HA cells cultivated to mid-exponential phase (OD_600nm_ 0.4) in low uracil medium, as described above. At different times before and during a heat shock from 75°C to 86°C, the culture was pulsed by adding either 4-thiouracil (4TU, 135 µM final concentration) or uracil (mock control, 45 + 135 µM; Fig. S1B). After 30 min, 7 mL culture samples were centrifuged for 8 min at 4,000 × *g* and 4°C, and the resulting pellets were frozen at −20°C.

Total RNA was extracted from the cell pellets using the hot-phenol procedure, which was followed by biotinylation with MTSEA-Biotin-XX (Biotium, USA, Fremont) as previously described (36, 37). Northern blotting was performed by subjecting 5.5 µg biotinylated RNA to a denaturing agarose RNA gel electrophoresis at 20 V, followed by a capillary transfer to a positively charged nylon membrane. Subsequently, biotinylated RNA was detected by DyLight 800-conjugated Streptavidin (Invitrogen, USA, Waltham) using the Odyssey CLx platform (LI-COR, USA, Lincoln) (36, 37). Streptavidin was stripped from the blot by pouring a boiling 1% SDS solution twice over the membrane. The blot was prehybridized with 50% formamide, 5× saline-sodium citrate (SSC; 750 mM NaCl, 75 mM sodium citrate, pH 7), 0.5% SDS, 5× Denhardt’s solution (1 mg mL^−1^ Ficoll 400, 1 mg mL^−1^ polyvinylpyrrolidone, 1 mg mL^−1^ bovine serum albumin fraction V) for 3 h at 30°C. For the detection of bulk steady-state rRNA, 10 pmol of fluorophore-coupled probes was added that target the 5′ end region of mature 16S or 23S rRNA (DY682-CTTATCCCTACCCCGATAGCGG or DY782-CGAGCATTTCGCTGCTTGCCG, respectively), followed by overnight incubation at 30°C. Blots were first washed with 2× SSC for 15 min, then with 1× SSC for 15 min at 37°C, and finally, rRNA was visualized using the Odyssey CLx platform (LI-COR, USA, Lincoln). Fluorescence signals were quantified with ImageJ 1.53e (38).

### Pulse labeling of neosynthesized protein

A bioorthogonal non-canonical amino acid tagging (BONCAT) approach was used for protein pulse-labeling experiments. *S. acidocaldarius* SK-1 × Thα-FLAG + Thβ−6*x*His + Thγ-HA cells were cultivated to mid-exponential phase (OD_600nm_ 0.33) in the absence of methionine, as described above. Next, a combined heat shock and pulse-labeling treatment was performed as described for RNA pulse labeling, with the exception that 1 mM L-azidohomoalanine (L-AHA) and 1 mM L-methionine were used as pulsing agent and mock control, respectively (Fig. S1B).

The BONCAT protocol employed in this study was slightly adapted from the one described for *Haloferax volcanii* (39). Briefly, proteins were extracted from the samples by boiling and were reduced using β-mercaptoethanol. Next, 133 µg total protein per sample was alkylated in fresh phosphate-buffered saline (PBS) with 0.2 M 2-chloroacetamide. L-AHA-containing proteins were labeled with Cy7 by strain-promoted azide-alkyne click-chemistry employing 1 µM dibenzylcyclooctyne-Cy7 (DBCO-Cy7) (Click Chemistry Tools, USA, Scottsdale). After performing a methanol-chloroform extraction, protein pellets were resuspended in 40 µL of 2× HU-buffer (100 mM Tris-HCl pH 6.8, 0.5 mM EDTA pH 8, 4 M Urea, 2.5% SDS) supplemented with 200 mM dithiothreitol (DTT). Protein aliquots of 5 µL were loaded on SDS-PAGE and separated at 180 V. Fluorescence signals of L-AHA-incorporated proteins were detected in-gel using an Odyssey CLx platform (LI-COR, USA, Lincoln). Steady-state bulk levels of protein were detected by Coomassie staining of the gel, and scanning was performed with the Gel Doc XR + System (Bio-Rad, USA, Hercules). Signals were quantified with ImageJ 1.53e (38).

## RESULTS

### Global transcriptomic and proteomic changes in response to heat shock

**Fig 1:**
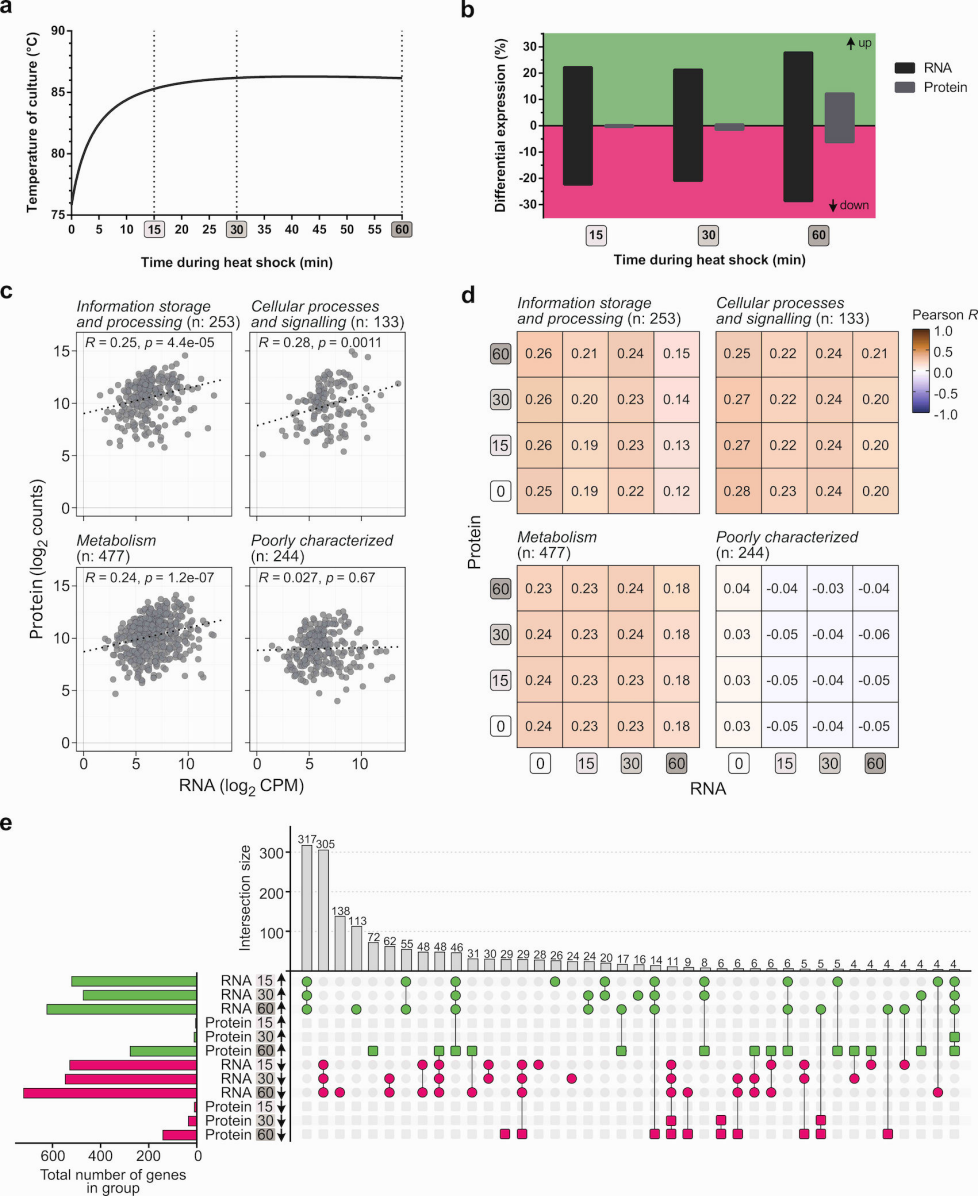
Global transcriptomic and proteomic changes in response to heat shock. (a) Temperature profile of the culture upon temperature shift from 75°C to 86°C, according to the heat shock setup described in reference (28) and Fig. S1. (b) Differential expression analysis depicting the percentage of RNA and protein with differential abundance at different heat shock time points, compared to levels at 75°C. (c) Correlation plot of the transcript and protein abundance at 75°C, subdivided according to the functional category of the respective gene. The Pearson correlation coefficient R is given for each category. (d) Correlation matrix plotting the Pearson correlation coefficient R for transcript and protein abundances at 75°C (= time point 0) and the heat shock time points (and the combinations thereof). (e) Plot visualizing the overlap (intersection) between the regulation groups (increased or decreased abundance at 15, 30, and 60 min after heat shock treatment at the RNA or protein level). The size of the regulation groups corresponds to the number of differentially expressed genes/proteins in that group (related to panel b).

When studying heat shock response, it is imperative to subject cells to a temperature change that elicits a stress response without affecting cellular integrity. At the optimal growth temperature of 75°C, the doubling time of *S. acidocaldarius* was previously determined to equal approximately 15 h during early to mid-exponential growth (28). During this growth phase, a rapid temperature shift from 75°C to 86°C (Fig. S1a) was shown to induce a regulatory response in *S. acidocaldarius*, as demonstrated by the upregulation of the thermosome Thα and Thβ subunits (Fig. S2a). This heat shock was shown not to impair cellular viability over the course of 60 min (Fig. S2b). With the aim of simulating how temperature gradients are sensed in the natural environment, we selected three time points after shifting cells to 86°C for further analysis (Fig. 1a). These represent conditions in which cells are still experiencing a temperature increase (15 min), in which a steady-state temperature has just been reached (30 min) and in which cells are subjected to a persistent heat shock condition (60 min).

Upon performing transcriptomic and proteomic analyses, it was confirmed that massive, global changes occur at the transcriptomic level in response to a temperature increase, while this response is much less pronounced at the proteomic level (Fig. 1b). Indeed, immediately after heat shock, 44.5% of all coding genes displayed a differential RNA abundance (1,047 of 2,351 genes), decreasing to 42.2% (991 genes) upon reaching a steady state temperature and increasing again to 56.4% at 60 min (1,325 genes). The fraction of proteins displaying differential abundance evolved from 0.7% (15 of 2,267 proteins) at 15 min to 2.0% (46 proteins) at 30 min and 18.4% (418 proteins) at 60 min of heat shock (Fig. 1b). While the number of genes with a higher and lower RNA level was approximately the same for the different time points, there was a bias toward a larger fraction of proteins having a higher and not lower abundance after 60 min (Fig. 1b).

The strongly differing numbers of transcripts and proteins with differential abundance suggest that there might be a limited overlap between transcript- and protein-level effects, which was assessed by a gene-by-gene correlation analysis (Fig. 1c and d). Indeed, a lack of correlation was confirmed not only by comparing the abundance of a particular transcript and its corresponding protein at 75°C and within the same heat shock time point but also when comparing RNA levels at, e.g., 15 or 30 min with protein levels at 60 min. A time-delayed reaction of the proteome in the same direction as the transcriptome does not seem to be a general pattern of the heat shock response in *S. acidocaldarius* (Fig. 1e). These observations suggest a prevalence of post-transcriptional and post-translational regulation upon heat shock in addition to the extensive transcriptional regulation that was observed.

### Functional categories of differentially expressed genes

**Fig 2:**
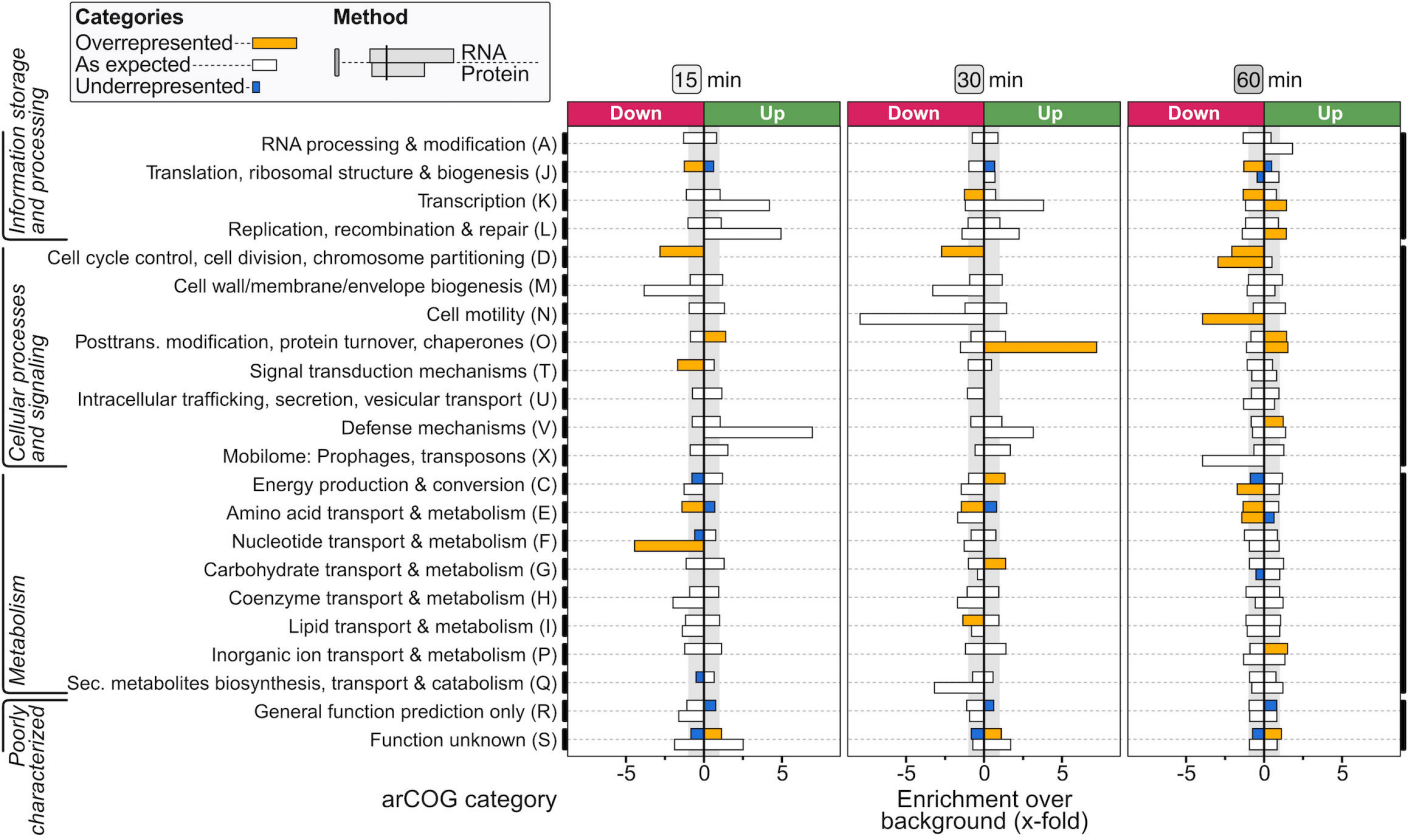
Gene set enrichment analysis of archaeal clusters of orthologous genes (arCOGs). Bars indicate the fold enrichment of an arCOG category (31) based on the total number of differentially expressed genes/proteins at that time point, taking into account the different number of genes and proteins in each arCOG category, i.e., if the percentage of differential expression in the arCOG category equals the average differential expression of all categories, enrichment equals zero. Bars are color coded according to statistically significant positive enrichment (“more upregulation or downregulation than on average,” overrepresentation) or statistically significant negative enrichment (“less upregulation or downregulation than on average,” underrepresentation) (*P* < 0.05). Note that one should be careful with the interpretation of the protein data at 15 and 30 min, given the low number of differentially produced proteins at these time points (see Fig. 1b).

With the aim of exploring the functional context of genes and proteins that display a heat shock responsive differential abundance, we performed a gene set enrichment analysis based on the arCOG classifications (31) (Fig. 2). This demonstrated that the transcriptional heat shock responsive regulation is not restricted to a limited set of functional categories, but affects diverse biological processes. For information storage and processing gene categories, such as transcription (arCOG K) and translation (arCOG J), a considerable transcriptional downregulation was observed at most time points (Fig. 2). In addition, an overrepresentation of downregulated transcripts and/or proteins was found within categories such as cell cycle control, cell division, chromosome partitioning (arCOG D), cell motility (arCOG N), and signal transduction mechanisms (arCOG T). In contrast, genes involved in post-translational modification and protein turnover (arCOG O) are significantly overrepresented in upregulated transcripts and proteins. Also, several metabolic pathways were affected, either positively or negatively, including pathways in nucleotide and amino acid metabolism (arCOG F and E), energy production (arCOG C), and carbohydrate metabolism (arCOG G) (Fig. 2). Certain key cellular processes affected by heat shock are discussed in more detail below. Others, including DNA replication, genome segregation, cell division, DNA repair, DNA import, and motility and biofilm formation, are described in Supplementary Results.

### Response of the heat shock protein machinery

**Fig 3:**
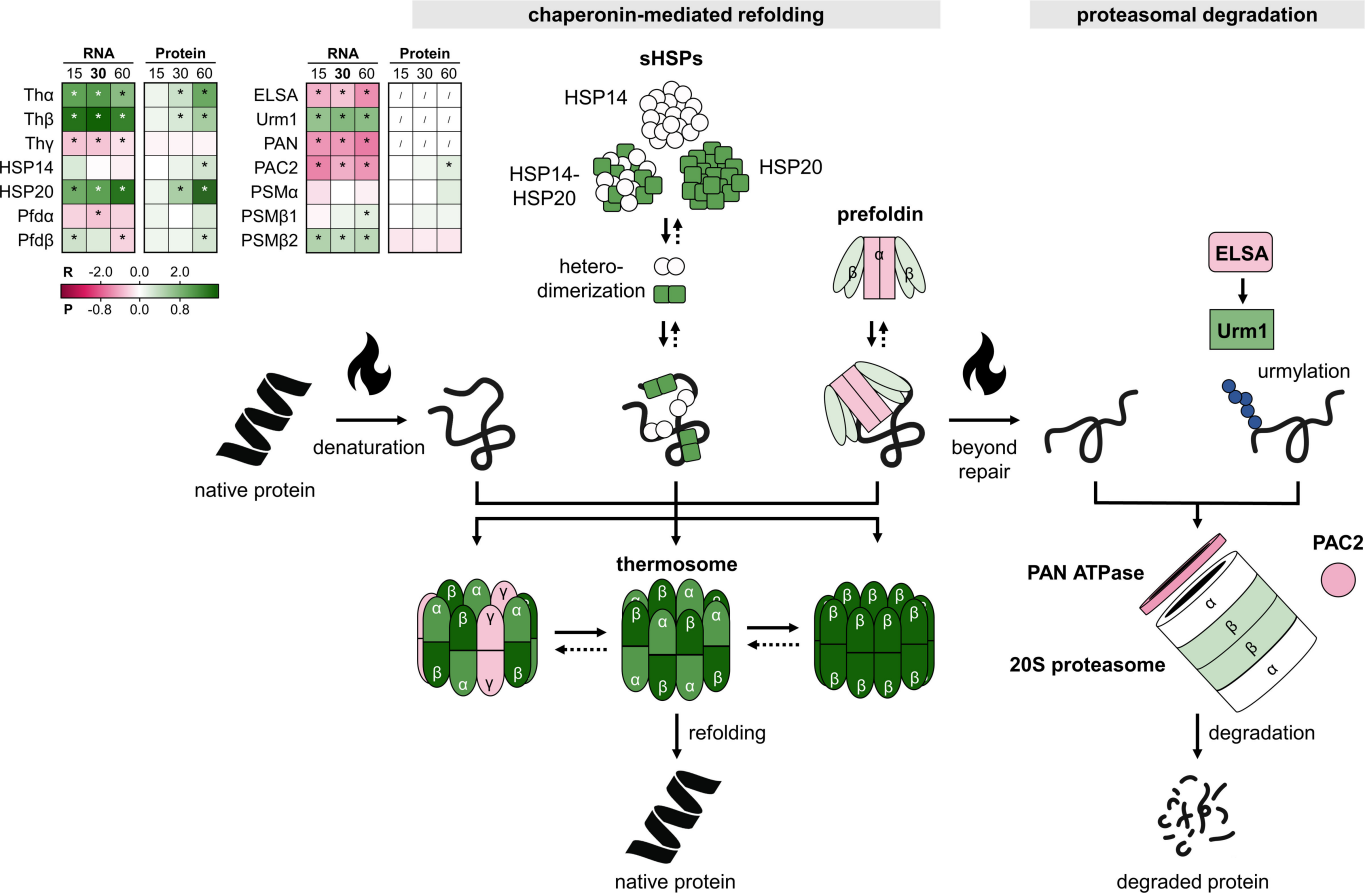
Heat shock responsive differential abundance of the chaperones, the heat shock proteins, and the proteasome. The inset table shows differential expression at the RNA (R) and protein (P) level at all time points (15, 30, and 60 min), color coded according to the log_2_FC value in the gradient below. * = significant (FDR/adj. *P*-value <0.05). / = not covered. The scheme on the right is colored according to differential expression at the RNA level at 30 min. The chaperone system consists of sHSPs (HSP14 and HSP20), prefoldin, and the thermosome. The first two bind denaturing proteins prevent their aggregation and shuttle them to the thermosome complex for active refolding and for which different subunit compositions were predicted (Thα, Thβ, and Thγ). Protein degradation is mediated by the 20S proteasome, which is composed of α-outer rings (PSMα) and an assembly of β-inner rings (PSMβ1 and PSMβ2), assisted by the proteasome assembly chaperone (PAC2) (40). The proteasome-activating nucleotidase (PAN) AAA^+^ ATPase is required for substrate unfolding before their translocation to the core proteasome and degradation (40). Archaeal-type urmylation, post-translational modification of protein substrates by Urm1/SAMP, can act as a signal for proteosome-mediated degradation by direct recognition of the modified protein by the PAN-ATPase and the core proteasome *in vitro* (40). Urm1/SAMP is activated by ELSA (40).

The most apparent upregulation was observed for the arCOG category O, comprising genes involved in post-translational modification, protein turnover, and chaperones, with a significant overrepresentation of upregulation on both the RNA and protein level at 60 min of heat shock treatment (Fig. 2). This category includes the HSP machinery and proteasomal degradation system as key elements in the preservation of the structural integrity of proteins (Fig. 3).

During exponential growth at the optimal growth temperature of 75°C, all HSPs were already highly expressed at both the RNA and protein level (Data set S2; Fig. S3). Indeed, the three thermosome subunits Thα, Thβ, and Thγ were found to be among the four most abundant proteins in the cell (Fig. S3), underlining the importance of this chaperone complex in proteome homeostasis and cellular physiology even in the absence of temperature stress. In response to heat shock, substantially increased transcript levels were observed for the Thα and Thβ subunits, followed by a slower increase in their protein levels, while the Thγ subunit was transcriptionally downregulated and its protein level was found not to be heat shock responsive (Fig. 3). This finding is in agreement with a western blot analysis of the thermosome-tagged strain (Fig. S2a) and previous findings in *S. acidocaldarius* and *Saccharolobus shibatae* (17, 28, 41). In addition, a steady increase was observed for HSP20, both at the transcript and protein level (Fig. 3). Moreover, of all proteins, HSP20 displayed the highest increase in abundance after 60 min of heat shock treatment (Data set S2; Fig. S3). Whereas no transcriptional regulation was observed for *hsp14*, its protein level was shown to increase upon heat shock (Fig. 3), suggesting that HSP14 production is regulated on the post-transcriptional level. Finally, heat shock responsiveness of prefoldin was shown not to be pronounced and only transient (Fig. 3).

When mis- or unfolded proteins cannot be rescued by the molecular chaperones, the proteasomal degradation machinery takes over to prevent protein aggregation. Our study revealed a mixed view for the response of the proteasome and its associated proteins to heat shock stress. Whereas some components were shown to be upregulated (e.g., a transcriptional increase was observed for the proteasome β subunits and Urm1), others were found to be downregulated [e.g., a steady decrease was observed for transcripts encoding the proteasome-activating nucleotidase (PAN) AAA^+^ ATPase, the proteasome assembly chaperone (PAC2) and ELSA] (Fig. 3). Possibly, urmylation modifications increase upon heat shock, although we are unable to detect Urm1 and ELSA in our proteomic analysis. Nevertheless, the lack of observing a considerable upregulation of essential proteasome components such as PAN ATPase and PAC2 suggests that chaperones can still compensate for the negative effects of high-temperature stress on proteins. Additional proteolytic support is not (yet) required for the heat shock conditions investigated in this study.

### Effects of heat shock on chromatin organization

**Fig 4:**
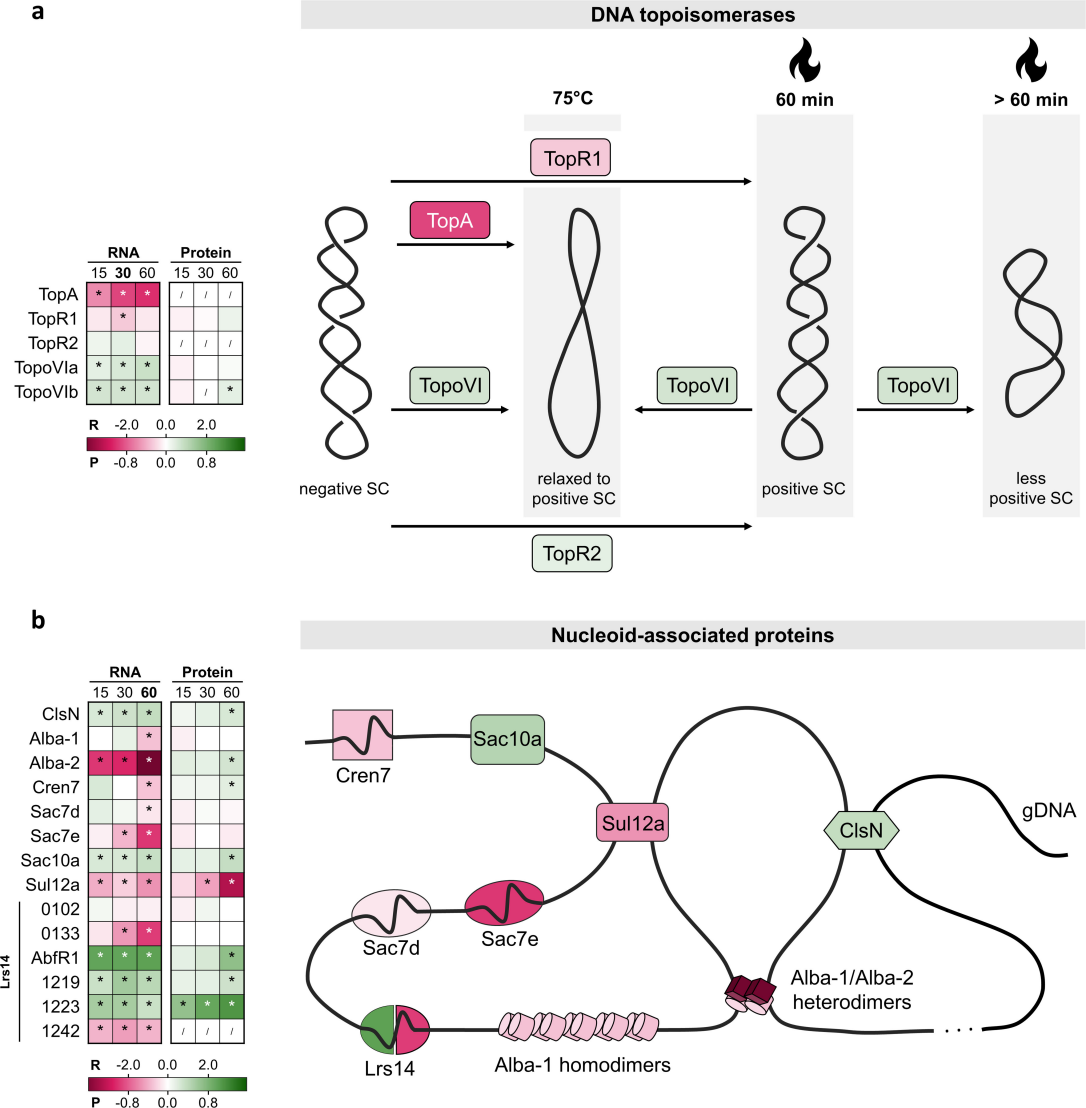
Heat shock responsive differential abundance of proteins involved in chromatin organization. The tables show the differential expression of the genes involved at the RNA (R) and protein (P) level at all time points after heat shock (15, 30, and 60 min), color coded according to the log_2_FC value in the gradient below. * = significant (FDR/adj. *P*-value <0.05). / = not covered. (**a) **DNA topoisomerases. The figure shows the proteins colored according to differential expression at the RNA level after 30 min and is inspired by references (10 – 12). Increased enzymatic activity of reverse gyrase TopR1 at higher temperatures (10, 45) causes an increase in linking number (positive supercoiling) immediately after heat shock in *S. islandicus* (10). As shown in references (11, 45), the number of TopR1 proteins is decreasing upon heat shock, which is associated with a gradual decrease in linking number and a new topological state of the DNA, adapted to the higher temperature after 60 min (10). TopoVI, is a type II topoisomerase able to relax both negative and positive supercoils at the optimal growth temperature, but is only active with the positively supercoiled substrate at 88°C, and activity is low (11, 12). (**b) **Nucleoid-associated proteins. The figure shows the proteins in their cellular context, colored according to differential expression at the RNA level after 60 min. In Sulfolobales, the set of NAPs is relatively extended and comprises an abundant class of proteins in the cell.

The chromatin of *S. acidocaldarius* has a relaxed to positively supercoiled topology (10, 42) and is further compacted and organized by a variety of nucleoid-associated proteins (NAPs) (43, 44). During the 1-h heat shock, we observed considerable changes in the expression level of genes encoding proteins involved in chromatin organization (Fig. 4).

DNA topoisomerases are involved in resolving topological stresses created by DNA-based processes (12). Here, topoisomerases were shown to display a differential transcriptional abundance in response to heat shock (Fig. 4a). At the protein level, this response was less pronounced. Whereas TopA levels had been undetectable in previous heat shock studies in Sulfolobales (11, 46), we observed a strong decrease in *topA* transcripts immediately after heat shock. Combined with the observation that TopA is efficient in relaxing negative supercoiling (47) and displays decreased activity at elevated temperatures (11), our results provide evidence that TopA is not required in homeostatic control of DNA topology after heat shock. In addition, transcript levels of reverse gyrase *topR1* were found to decrease within 30 min of heat shock implementation, while *topR2* was shown not to be heat shock responsive (Fig. 4a). These results are in line with the proposed model of a TopR1/TopoVI-induced increase in positive DNA supercoiling upon heat stress and a subsequent decrease in linking number (10 – 12). In this context, it is not an unexpected finding that TopR1 is transcriptionally regulated in response to heat shock (Fig. 4a). In contrast, TopR2 has been proposed to be involved in DNA replication and repair (12) (Supplementary Results) and thus to be the most important reverse gyrase at optimal growth temperature, which is confirmed by a viability analysis of deletion strains in *S. islandicus* (48). Finally, both TopoVI subunits displayed an increased abundance at the transcript level and TopoVI subunit B also at the protein level (Fig. 4a). Given that subunit B is the limiting subunit in functional TopoVI and that its activity is low at elevated temperatures (11), it can be assumed that an increased abundance of TopoVI is required to decrease the linking number of the positively supercoiled DNA after about 60 min as a result of increased TopR1 activity (10, 11).

By interacting with DNA in a non-sequence specific manner, NAPs combine an architectural role with a global gene regulatory role (through the differential organization of the chromosome) and/or a specific gene regulatory role (e.g., through binding transcription-factor binding sites) (43, 49). The macro-level chromosome organizer coalescin (ClsN) (50) was found to be slightly more abundant upon heat shock, both on the transcriptional level (at all time points) and the protein level (after 60 min) (Fig. 4b). Focusing on the two Alba paralogs, we observed a late transcriptional downregulation of *alba-1* (after 60 min). In contrast, the *alba-2* RNA level displayed a strong decrease at all time points during heat shock, while remarkably, its protein level was increased after 60 min (Fig. 4b). These observations suggest that there might not be an increase in Alba-1-Alba-2 heterodimers (51), but Alba-2-regulated chromosome organization plays a role in heat shock response.

All small NAPs, except *sac10a*, displayed a decreased abundance at the RNA level upon heat shock, although this was not reflected on a protein level (Fig. 4b). In contrast, Cren7 and Sac10a displayed a slight but significant increase in their protein levels.

The Lrs14 family of NAPs was previously shown to help controlling DNA topology after heat shock and to stabilize DNA against thermodenaturation by introduction of local positive supercoiling (52, 53). Upon heat shock, it was observed that five out of six members of this family were differentially expressed, with three members displaying an increase in transcript and protein levels (Fig. 4b). Moreover, it is notable that *Saci_1223* is one of the most upregulated proteins at all time points and AbfR1 at 60 min (Data set S2).

### Effects of heat shock on the functioning of the transcription process

Our results demonstrate considerable changes in transcript and protein abundance for many cellular processes in response to heat shock. However, the observation of differential abundance does not necessarily result from changes in transcriptional or translational activity. Instead, it could also be the consequence of changes in RNA or protein stability, thereby affecting its degradation. To investigate this possibility, we analyzed the impact of heat shock treatment on the overall transcriptional and translational activity in *S. acidocaldarius* using a pulse-labeling approach.

**Fig 5:**
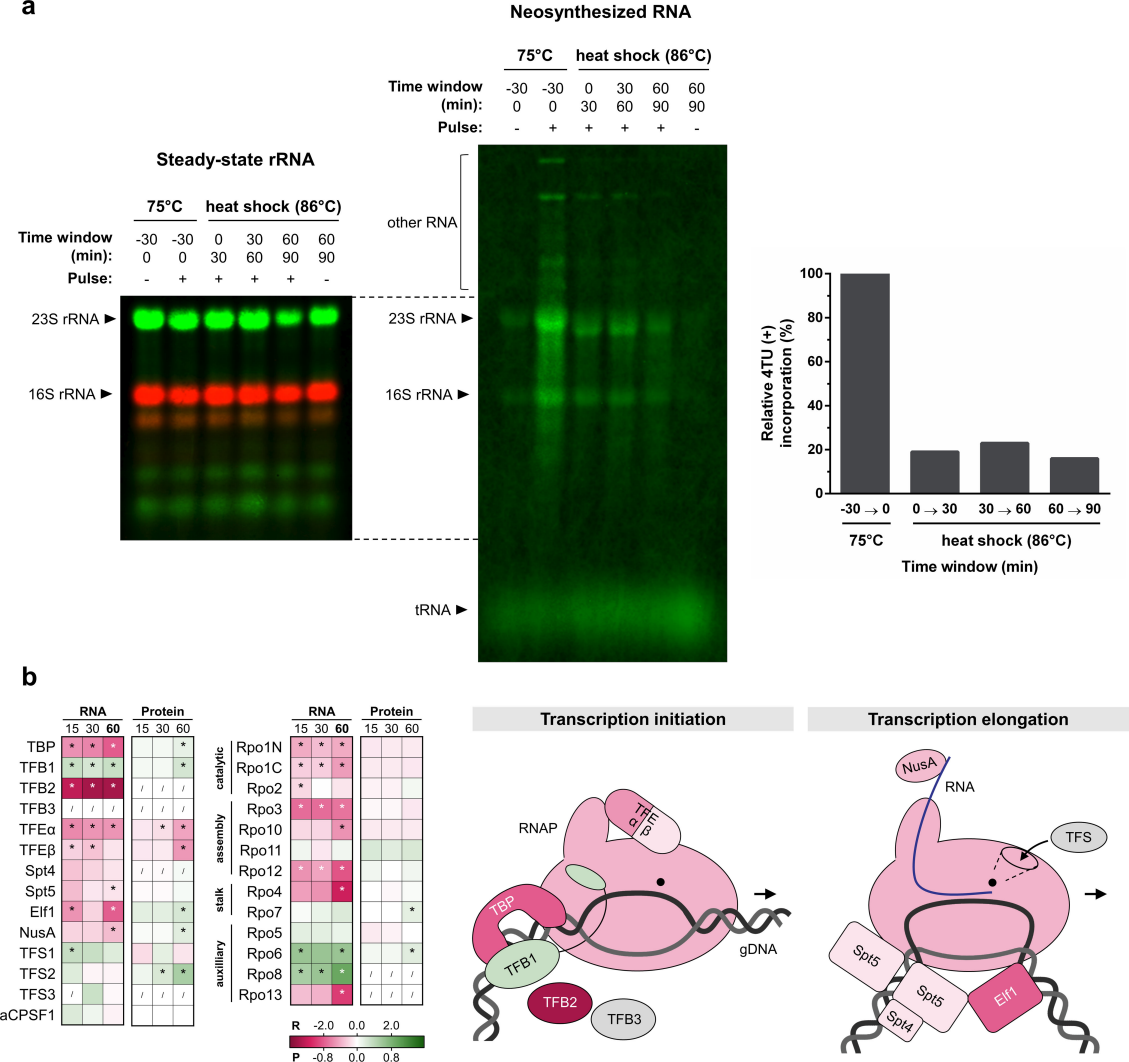
Impact of heat shock on transcription. (a) Pulse labeling of neosynthesized RNA. SK-1 Thα-FLAG + Thβ−6xHis + Thγ-HA cultures were pulsed, both at 75°C and upon an 86°C heat shock for 30-min time windows by addition of an excess of nucleotide analog 4TU (+) or uracil (−) as mock control. 4TU was incorporated into neosynthesized RNA, and 4TU-labeled RNA was biotinylated in the total pool of RNA. Total RNA was analyzed by Northern blotting. Bulk steady-state rRNA was detected by DY682- or DY782-coupled probes targeting the 5′ end region of mature 16S or 23S rRNA, serving as a loading control (left panel). 4TU-labeled RNA was detected by DyLight800-conjugated streptavidin (central panel). The incorporation of 4TU into 16S or 23S rRNA was quantified by first subtracting the background signal in the uracil-mock control and dividing this by the signal of the steady-state rRNA. Relative 4TU-incorporations upon heat shock were calculated relative to incorporation at 75°C, and the average of 16S and 23S rRNA was determined as a ratio to express the relative transcriptional activity upon heat shock (right panel). (**b)** Differential expression of the transcription machinery upon heat shock. The inset table shows differential expression at the RNA (R) and protein (P) level at all time points (15, 30, and 60 min), color coded according to the log_2_FC value in the gradient below. * = significant (FDR/adj. *P*-value <0.05). / = not covered. The graphical scheme on the right is colored according to differential expression at the RNA level at 60 min and is adapted from reference (54). Transcription is initiated by binding of the TATA-binding protein (TBP) and transcription factor B1 (TFB1) to the TATA-box and B-recognition element (BRE) of the promoter, respectively. The RNA polymerase (RNAP/Rpo) is recruited, transcription factor E (TFE) assists in DNA melting, and the pre-initiation complex is formed (54, 55). After initiation, TFE-binding to RNAP is replaced by binding of elongation factors Spt4/5, and the elongation ensues with the help of Elf1 and NusA (54, 55). Transcription factor S (TFS) is involved in RNA cleavage and induction of resynthesis in stalled elongation complexes (56). Transcription termination occurs at poly(U) stretches or is assisted by the archaeal termination factor aCPSF1 (57, 58).

For the analysis of transcriptional activity, the uracil analog 4TU (36, 37) was allowed to follow the *de novo* synthesis of RNAs before and during heat shock (Fig. 5a; Fig. S4). The major transcriptional activity of the cell comprises rRNA synthesis, and thus, as expected, 16S and 23S rRNA were the major species of neosynthesized RNA, both during growth at 75°C and during heat shock. In addition, several larger RNA species were neosynthesized within a 30-min time frame, which are hypothesized to be mRNA species with a high abundance and/or turnover rate, given that they are not hybridized by rRNA probes (Fig. S4a). Immediately after heat shock, a drastic decrease was observed for 4TU incorporation in rRNAs down to 20%–40% of the initial levels at 75°C. The low level of neosynthesized rRNAs was maintained for at least 2 h after heat shock (Fig. 5a; Fig. S4b), suggesting that heat shock causes the transcriptional activity to decline quickly and persistently.

A decline in transcriptional activity is also supported by a downregulation for most of the individual components of the basal transcription machinery on the transcriptional level, as determined by the RNA-seq analysis (Fig. 5b). For example, immediately after heat shock, transcript levels were found to be decreased significantly for TATA-binding protein (TBP), for the catalytic, assembly, and stalk subunits of RNA polymerase (RNAP) and for the α and β subunits of basal transcription factor TFE (Fig. 5b). However, on the protein level, differential abundance and regulatory trends were less pronounced, and only significant downregulation was observed for TFEα and β, associated with a slight increase in TBP protein levels. In contrast, TFB1, which is considered as the housekeeping TFB in *S. acidocaldarius*, was slightly upregulated on both the transcript and protein level, while the alternative TFB-type factor TFB2, of which the function remains enigmatic (54), displayed a strong transcriptional downregulation and was undetectable at the protein level.

Transcription elongation factors also exhibited a trend toward transcriptional downregulation upon heat shock, associated with slight increase in their protein levels (Fig. 5b). For TFS2, a considerable upregulation was observed already after 30 min.

### Effects of heat shock on the functioning of the translation process

**Fig 6:**
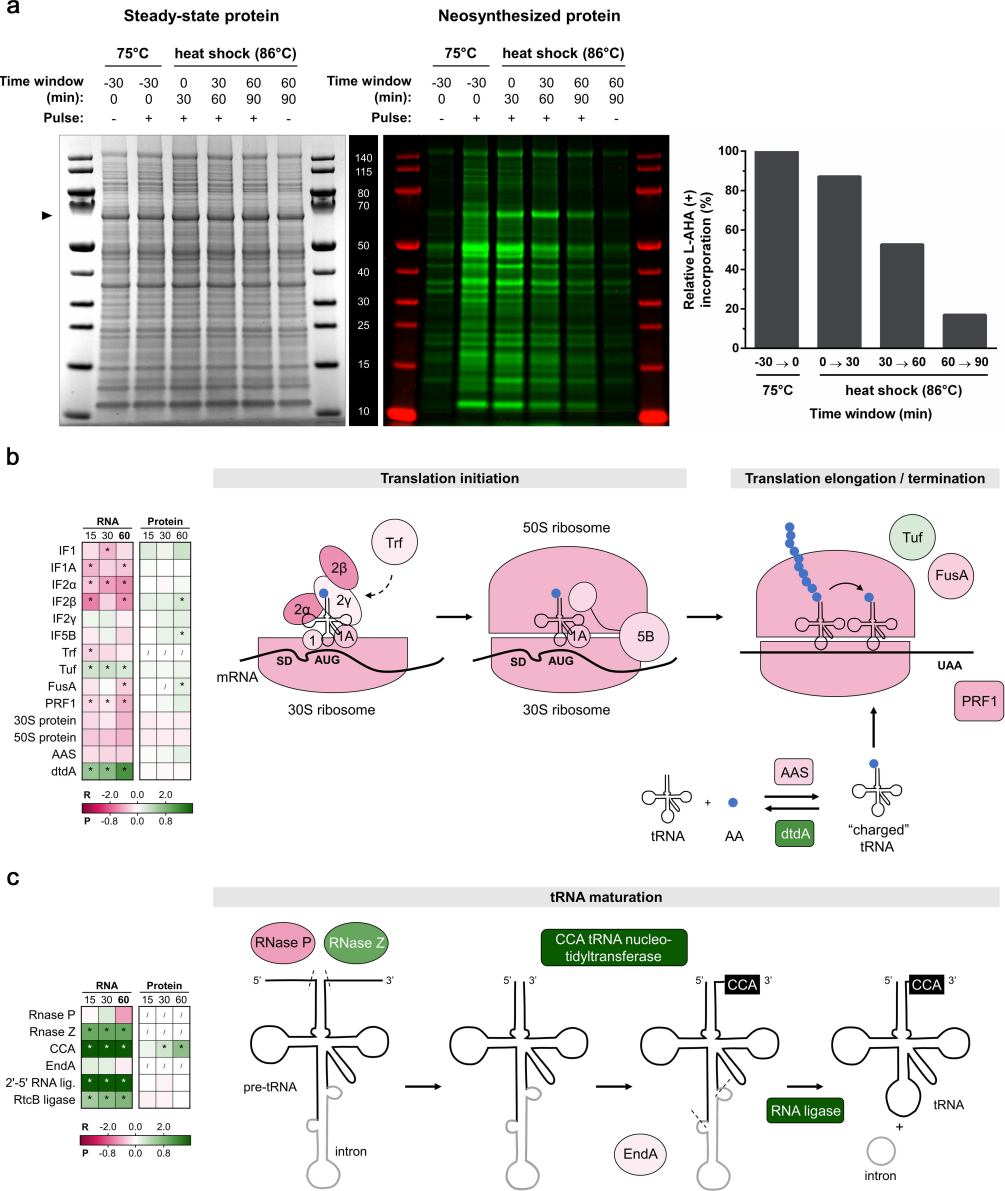
Impact of heat shock on translation and the translation machinery. (a) Pulse labeling of neosynthesized protein. *S. acidocaldarius* SK-1 Thα-FLAG + Thβ−6xHis + Thγ-HA was pulsed at 75°C and upon heat shock for 30-min time windows by addition of an excess of amino acid analog L-AHA (+) or methionine (−) as mock control. Total proteins were extracted, reduced, and alkylated. L-AHA-incorporated proteins in the total protein pool were labeled with Cy7 by strain-promoted azide-alkyne click-chemistry. Total proteins were separated by SDS-PAGE. Bulk, steady-state levels of protein were detected by Coomassie staining (left panel), serving as a loading control. L-AHA-incorporated proteins were detected in-gel by Cy7-fluorescence detection (central panel) and quantified with respect to total protein (right panel). (**b-c)** Differential expression of the translation machinery and tRNA maturation, respectively, upon heat shock. The inset tables show differential expression at the RNA (R) and protein (P) level at all time points (15, 30, and 60 min), color coded according to the log_2_FC value in the gradient below. * = significant (FDR/adj. *P*-value <0.05). / = not covered. The schemes on the right are color coded according to the differential expression at the RNA level at 60 min. (**b)** Translation initiation factors IF1 and IF1A stimulate the binding of the IF2 to the initiator tRNA and the small 30S ribosomal subunit, with IF2 being crucial for start codon recognition (59). Translation recovery factor (Trf) triggers the release of IF2 of the mRNA (60). Upon IF1 and IF2 departure, IF5B recruits the large 50S ribosomal subunit (59). L-aminoacyl-tRNA synthetases (AASs) are involved in ligating free tRNA to amino acid residues, forming “charged” L-aminoacyl-tRNA. In contrast, D-aminoacyl-tRNA deacylase (dtdA) is involved in recycling toxic D-aminoacyl-tRNAs to D-amino acids and free tRNAs. Upon elongation, the translation elongation factor 1α (Tuf) promotes the binding of L-aminoacyl-tRNA to the A-site of the ribosome, and elongation factor 2 (FusA) catalyzes the ribosomal translocation step. The polypeptide chain release factor (PRF1) assists in translation termination. For the AASs and ribosomal proteins, the color code corresponds to the average log_2_FC value of all genes/proteins classified as such. Scheme is adapted from references (59, 61). (**c)** Generation of mature tRNA is initiated by the removal of the 5′ leader and 3′ trailer from pre-tRNA by RNase P and RNase Z, respectively (61). CCA-tRNA-nucleotidyltransferase builds the 3′-terminal CCA sequence of tRNAs (61, 62). About half of crenarchaeal tRNA genes contain introns, which are removed by an RNA splicing endonuclease EndA, followed by ligation of the splice products by an RNA ligase and finally yielding a mature tRNA (61, 63). Schemes adapted from references (59, 61).

Pulse-labeling experiments were also performed to map *de novo* translation by making use of the methionine analog L-AHA (39). Overall translational activity decreased after the temperature shift (Fig. 6a). However, this heat shock responsive decline proceeded linearly and at a slower pace than the decline in transcriptional activity. Contrary to this general trend, a small subset of abundant proteins was produced at higher levels upon heat shock. For example, this was the case for a protein represented by a noticeable band at 60 kDa (Fig. 6a), which could be hypothesized to represent the thermosome Thα and/or Thβ subunit, as was also observed by western blotting in the thermosome-tagged strain (Fig. S2a).

As observed for the basal transcription machinery, most components of the translation initiation machinery were found to be transcriptionally downregulated upon heat shock, while this regulatory trend was not carried on at the protein level (Fig. 6b). Conversely, IF2β and IF5B protein levels were increased at the 60-min time point. With respect to translational elongation, Tuf displayed a slightly increased RNA abundance, while FusA displayed a slightly decreased RNA abundance and increased protein abundance. The translational termination factor PRF1 was transcriptionally downregulated. In addition, the RNA levels of most L-aminoacyl-tRNA synthetases (AASs), responsible for coupling of the correct amino acid to tRNAs, decreased (Data set S2). In contrast, D-aminoacyl-tRNA deacylase, involved in recycling toxic D-aminoacyl-tRNA, was strongly transcriptionally induced upon heat shock (Fig. 6b).

Furthermore, considerable changes were observed in the expression of several components of tRNA and rRNA maturation pathways (Fig. 6c). More specifically, the operon consisting of CCA tRNA nucleotidyltransferase, which builds and repairs the 3′-terminal CCA sequence of tRNAs, and a 2*'*−5*'* RNA ligase were among the most highly transcriptionally upregulated genes upon heat shock. CCA tRNA nucleotidyltransferase was also strongly upregulated at the protein level after 30 and 60 min. In addition, also RNase Z and RNA ligase RtcB are strongly upregulated at the RNA level (Fig. 6c). Additionally, many enzymes involved in tRNA modification are differentially expressed after heat shock, albeit to a smaller extent (Data set S2). These observations indicate that although translational activity itself is slowed down upon heat shock, auxiliary processes that ensure translational fidelity and RNA processing are maintained or even upregulated.

### Search for putative heat shock responsive *cis*-regulatory elements

**Fig 7:**
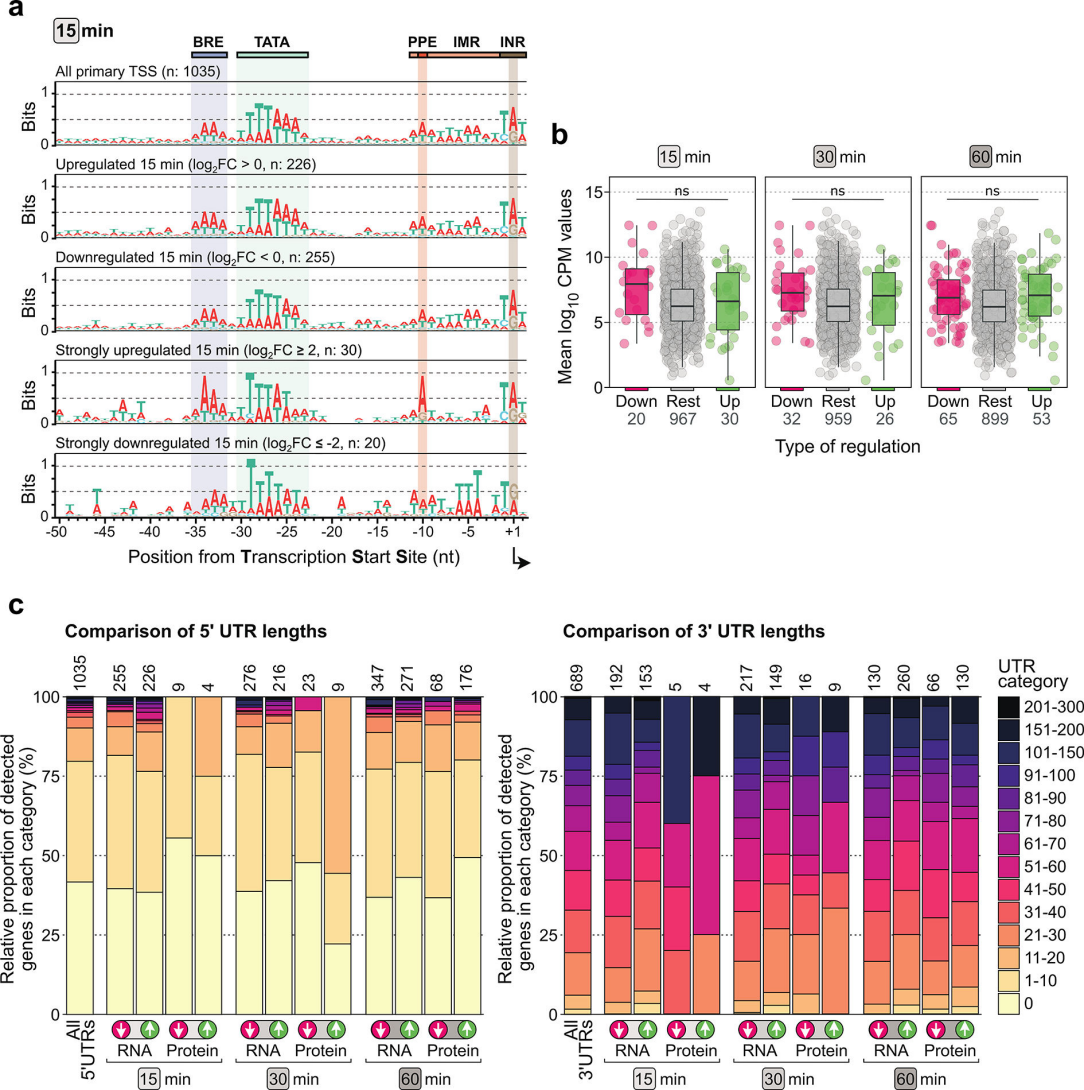
Search for putative heat shock responsive *cis*-regulatory elements in promoter and UTR regions. (a) Promoter motifs of all primary transcripts and subsets of regulation groups at 15 min after heat shock. PPE, proximal promoter element; IMR, initially melted region; INR, initiator element. (**b)** The abundance of a given transcript at 75°C is plotted, subdivided according to the regulation groups at the different heat shock time points. Rest = not differentially expressed upon heat shock. Up and down = strong up- or downregulation (log_2_FC ≥2 or ≤−2) after heat shock, respectively. A Welch’s *t*-test was used to assess differences between groups; significance level “ns” indicates *P*-values ≥0.01. High or low transcript levels at 75°C are not a prerequisite for strong up- or downregulation upon heat shock. (**c)** Bar plot showing the proportion of UTR-lengths (5′ UTR on the left panel and 3′ UTR on the right panel) for all genes in standard growth conditions (left bar) and for the differentially expressed transcripts/proteins at the different heat shock time points. Information on 5′ UTR length was retrieved from reference (33) and 3′ UTR length from reference (64).

In order to infer a putative protein-binding recognition motif for a presumptive heat shock transcription factor, we screened promotor regions of transcripts with the most significant differential expression. Analysis in a position-unspecific manner did not yield any significantly enriched motifs in any of the regulation groups, which is furthermore supported by the plotted consensus motifs in these promoter regions (Fig. 7a; Fig. S5). Despite the absence of a transcription factor-binding motif, it was apparent that strongly upregulated transcripts (especially at 15 and 30 min during heat shock) harbor more highly conserved B-recognition element (BRE) and proximal promoter element (PPE) promoter elements (Fig. 7a; Fig. S5). This subset of genes did not have a significantly higher constitutive level of transcriptional expression in optimal growth conditions at 75°C (Fig. 7b). Therefore, a strong BRE and PPE promoter element seems to be an adaptation of highly induced heat shock responsive genes and is linked to the regulation and not to basal transcription, possibly through the action of the TFB and/or TFE transcription initiation factors.

In the next step, we assessed whether a correlation exists between 5′ and 3′-UTR lengths of mRNAs and their differential abundance on RNA or protein level (Fig. 7c). Most transcripts in *S. acidocaldarius* are leaderless and lack a 5′-UTR (33, 65). However, it could be hypothesized to be a hot-spot region for temperature-responsive regulatory elements, as found in bacterial systems [e.g., RNA thermosensor element in the σ^32^ 5′-UTR (66, 67)]. No correlation was found for 3′-UTRs on both the RNA and protein level and for 5′-UTRs on the RNA level (Fig. 7c). On the protein level, however, nine proteins originating from transcripts with a 5′-UTR displayed a higher abundance at 30 min after heat shock (Fig. 7c). It should be noted that, out of these nine, five have a 5′-UTR length between 11 and 20 nucleotides (nts), as opposed to 10.5% for all genes. Notably, these five transcripts encode the thermosome Thα subunit (*Saci_1401*), thermosome Thβ subunit (*Saci_0666*), HSP20 (*Saci_0922*), an Lrs14-like DNA binding protein (*Saci_1223*), and TFS2 (*Saci_1587*). Among these, several fulfill key functions in heat shock response, as described above.

### Effects of heat shock on post-transcriptional and post-translational modifications

Our results point toward considerable post-transcriptional and post-translational regulation in response to heat shock. In order to rapidly modify the activity, function, and stability of an RNA species or protein, a universally conserved mechanism consists of the addition of post-transcriptional and or post-translational modifications (68). It has previously been shown that ribose methylation of rRNA and N^4^-acetylcytidine modification of RNA increases at higher cultivation temperatures of archaea, which might play a role in structural stabilization (69, 70).

Of all post-translational modifications, reversible protein phosphorylation is the most prevalent in *S. acidocaldarius* (71, 72). Upon heat shock, we observed that many kinases are differentially expressed (Data set S2). Furthermore, an immediate transcriptional upregulation of the phosphatases *Saci-PTP* and a downregulation of *Saci-PP2A*3 were noted. Various protein kinases and phosphorylated transcriptional regulators have been characterized thus far in Sulfolobales involved in a wide range of cellular processes (44, 71), including chromosome organization, motility and biofilm formation (53, 73), fatty acid metabolism (74), and homologous recombination (75). Each of the two protein phosphatases of *S. acidocaldarius* has unique dephosphorylation target residues (72). Therefore, small changes in phosphorylation status upon heat shock could lead to major consequences for different cellular processes.

Methylation of lysine residues is also a prevalent post-translational modification in thermophiles: it is proposed as a strategy to improve protein thermostability and to infer an additional layer of regulation (76). Methylations occur on proteins belonging to all arCOG categories, including many chromatin-associated proteins in *S. islandicus* (such as Alba-1, Alba-2, Cren7, and the Sac7d homolog) (76) and replication- and transcription-associated proteins. Methionine adenosyltransferase, involved in the generation of the major methyl donor, is transcriptionally downregulated upon heat shock yet shows increased protein levels (Data set S2). In addition, the lysine methyltransferase encoded by *Saci_1539* was found to be transcriptionally downregulated.

Protein glycosylation, for example of the S-layer subunit SlaA, also contributes to thermal stability by limiting peptide backbone flexibility (68). Several enzymes involved in this pathway were found to display an increased abundance upon heat shock at the transcript and/or protein level (Data set S2). Examples are the oligosaccharyl transferase aglB archaeal glycosylation enzyme 3, a predicted phosphoglucomutase/phosphor mannomutase and a predicted 3-hexulose-6-phosphate synthase. Therefore, besides the observed response at the transcriptome and proteome level, our results demonstrate that heat shock induces major changes at the post-transcriptional and post-translational level.

## DISCUSSION

In this work, we reveal that a temperature increase above the optimal growth temperature causes large changes in differential abundance of transcripts and proteins in *S. acidocaldarius*, with a fast and immediate response on the RNA level and a less pronounced and slower response on the protein level. Clearly, heat shock induces major effects on information processing, although it can be anticipated that this is not only the result of regulation taking place, but also of RNA and protein denaturation caused by a lack of thermostability and of changes in the synthesis and degradation rates (77), both of which are enzymatically catalyzed and thus inherently dependent on temperature. The use of a pulse-labeling approach enabled us to visualize RNA and protein neosynthesis and to demonstrate that global transcription and translation activity decreases in response to heat shock, albeit with different response dynamics.

The fast decline in transcriptional activity does not align with a lowered protein abundance of the basal transcription machinery consisting of the RNAP and general transcription initiation factors. The exception to this observation is the initiation factor TFE, for which both subunits are significantly downregulated on the transcript and protein level. A heat shock responsive depletion of TFE subunit α was also previously observed in the related species *Sa. solfataricus* (78). Notwithstanding temperature-dependent effects on the activity of all components of the basal transcription machinery, these results point to a key role for TFE, which optimizes transcription initiation by stabilizing the open transcription bubble (79, 80) by reducing the rate of transcription initiation upon heat stress. It could be hypothesized that the organism maintains a similar level of protein availability of other components of the transcription machinery to enable a fast recovery after the heat shock condition is relieved. In contrast to components of the transcription preinitiation complex, a considerable upregulation was observed on the protein level for TFS2 in response to heat shock. This transcription elongation factor is involved in RNA cleavage and restarting transcription within stalled elongation complexes (56).

In contrast to transcription, translational activity decreases slowly but consistently in response to heat shock. This is accompanied by a transcriptional downregulation of translation initiation and elongation factors and most L-aminoacyl-tRNA synthetases. Together with the finding that heat shock causes an immediate decrease in levels of neosynthesized 16S and 23S rRNA and the decrease in the abundance of many 30S and 50S ribosomal proteins, this demonstrated that there is a change in the translational capacity. In striking contrast, a considerable increase was observed for the expression of genes involved in tRNA and rRNA maturation. Whereas RNA-seq did not allow reliable quantification of tRNA, it has previously been shown that the tRNA pool was shifted upon stress conditions in yeast (81).

Despite lowered transcriptional and translational activities, the pulse-labeling experiments clearly indicated that neosynthesis is still taking place under the heat shock conditions chosen in these experiments. Consequently, it is likely that true regulatory events are taking place, especially for genes displaying a higher transcript and/or protein abundance upon heat shock. Changes at the transcriptomic level are not clearly correlated to those on the proteomic level; moreover, it is remarkable that despite the lower number of genes with a significantly lower or higher abundance at the protein level, the RNA level of many of these genes remained unchanged. Such a lack of correlation between transcriptomic and proteomic dynamics has also been observed in *S. acidocaldarius* for other stress conditions such as nutrient limitation (82) and was also found for heat shock response in bacteria (83, 84). It suggests a prevalence of post-transcriptional and/or post-translational regulatory events in addition to the extensive transcriptional regulation observed upon heat shock.

In contrast to eukaryotic and bacterial systems, as well as to the euryarchaeal *P. furiosus* and *A. fulgidus* (23, 24, 26), it seems unlikely that *S. acidocaldarius* harbors a classical heat shock transcription factor that is responsible for the transcriptional upregulation of important heat shock responsive genes such as HSPs. This is corroborated by the absence of a conserved motif in heat shock regulon promoters and refutes previous hypotheses that heat-shock-induced transcription factors might play such a role in *S. acidocaldarius* (28). Instead, a strong transcriptional upregulation might be mediated by TFB, and possibly TFE recruitment, given the highly conserved BRE and PPE signatures. Considering the downregulation of TFE during heat shock, a strong PPE element could possibly discriminate between genes that require strong induction and the others.

Alternatively, given the connection between chromatin organization and transcriptional activity in *Sulfolobales* (43), the observed dynamic regulation of DNA supercoiling and structuring could be hypothesized to play an important role in heat shock responsive global regulation and could underly the observed gene regulation on the transcriptional level. Indeed, our findings confirm the TopR1-model of increased DNA-positive supercoiling upon heat shock (10 – 12). Furthermore, the chromosome of *S. acidocaldarius* and other Crenarchaeota is compacted by a large array of small bacterial-like NAPs (43). NAPs seem to play an important role in preventing DNA denaturation at heat shock temperatures as observed and proposed for other hyperthermophilic archaea (85); however, they might also play a role in temperature-responsive global gene regulation. Previously, it was shown that cold shock leads to altered short- and long-range interactions and an associated decreased ClsN occupancy and transcriptional activity in the chromosome of *S. islandicus* (50). Here, it is shown that heat shock induces altered levels of various chromatin proteins, not only ClsN, but also classical NAPs and Lrs14-family proteins, with the latter type also being involved in transcription regulation (43, 52, 53, 85). In conclusion, we hypothesize that in contrast to histone-harboring Euryarchaeota that have heat shock transcription factors such as Phr, Sulfolobales and other histone-lacking thermophilic archaea employ an evolutionary ancient mechanism relying on temperature-responsive changes in DNA organization and compaction, induced by the action of NAPs, in combination with a considerable portion of post-transcriptional and post-translational regulation.

## Data Availability

RNA-seq data have been deposited in the European Nucleotide Archive at EMBL-EBI under accession number PRJEB57647, and MS data have been deposited to the ProteomeXchange Consortium via the PRIDE (86) partner repository with the data set identifier PXD038744. Convenient, easily accessible lists of genes of pathways with their corresponding expression levels are available in Data set S2. Code for all bioinformatic analyses is available via Github.

## SUPPLEMENTAL MATERIAL

Data Set S1 mbio.03593-22-s0001.xlsx Overview of cultures and samples in omic experiments, RNA-seq processing parameters, and sequencing stats.

Data Set S2 mbio.03593-22-s0002.xlsx Overview of RNA-seq and MS data.

Figure S1 mbio.03593-22-s0003.tif Technical set-up and experimental design of the heat shock sampling for the omics and pulse-labelings.

Figure S2 mbio.03593-22-s0004.tif Validation of induction of heat shock response in the SK-1 Thα-FLAG+Thβ-6xHis+Thγ-HA control strain in the heat shock setup of RNA-seq and MS.

Figure S3 mbio.03593-22-s0005.tif Top 30 of the most abundant transcripts and proteins in exponentially growing *S. acidocaldarius* at 75°C and upon heat shock.

Figure S4 mbio.03593-22-s0006.tif Pulse-labeling of neosynthesized RNA in exponentially growing *S. acidocaldarius* at 75°C and upon heat shock, extended set of data.

Figure S5 mbio.03593-22-s0007.tif Extended motif of the promoter and 5' end of primary transcripts and subsets of regulation groups.

Supplemental Methods mbio.03593-22-s0008.pdf Construction of the tagged thermosome strain, analysis of cellular viability by plating, western blotting, RNA sequencing, and TMT-labeled liquid chromatography-tandem-mass-spectrometry.

Supplemental Results mbio.03593-22-s0009.pdf Effects of heat shock on DNA replication, genome segregation and cell division, DNA repair, DNA import and motility, and biofilm formation.

Table S1 mbio.03593-22-s0010.pdf Microbial strains used in this study.

ASM does not own the copyrights to Supplemental Material that may be linked to, or accessed through, an article. The authors have granted ASM a non-exclusive, world-wide license to publish the Supplemental Material files. Please contact the corresponding author directly for reuse.
