# Supplementary figures and images for: Transcriptional and translational dynamics underlying heat shock response in the thermophilic crenarchaeon Sulfolobus acidocaldarius
Source: mBio. 2023 Aug 29;14(5):e03593-22. doi: 10.1128/mbio.03593-22 (PMC10653856; doi:10.1128/mbio.03593-22)

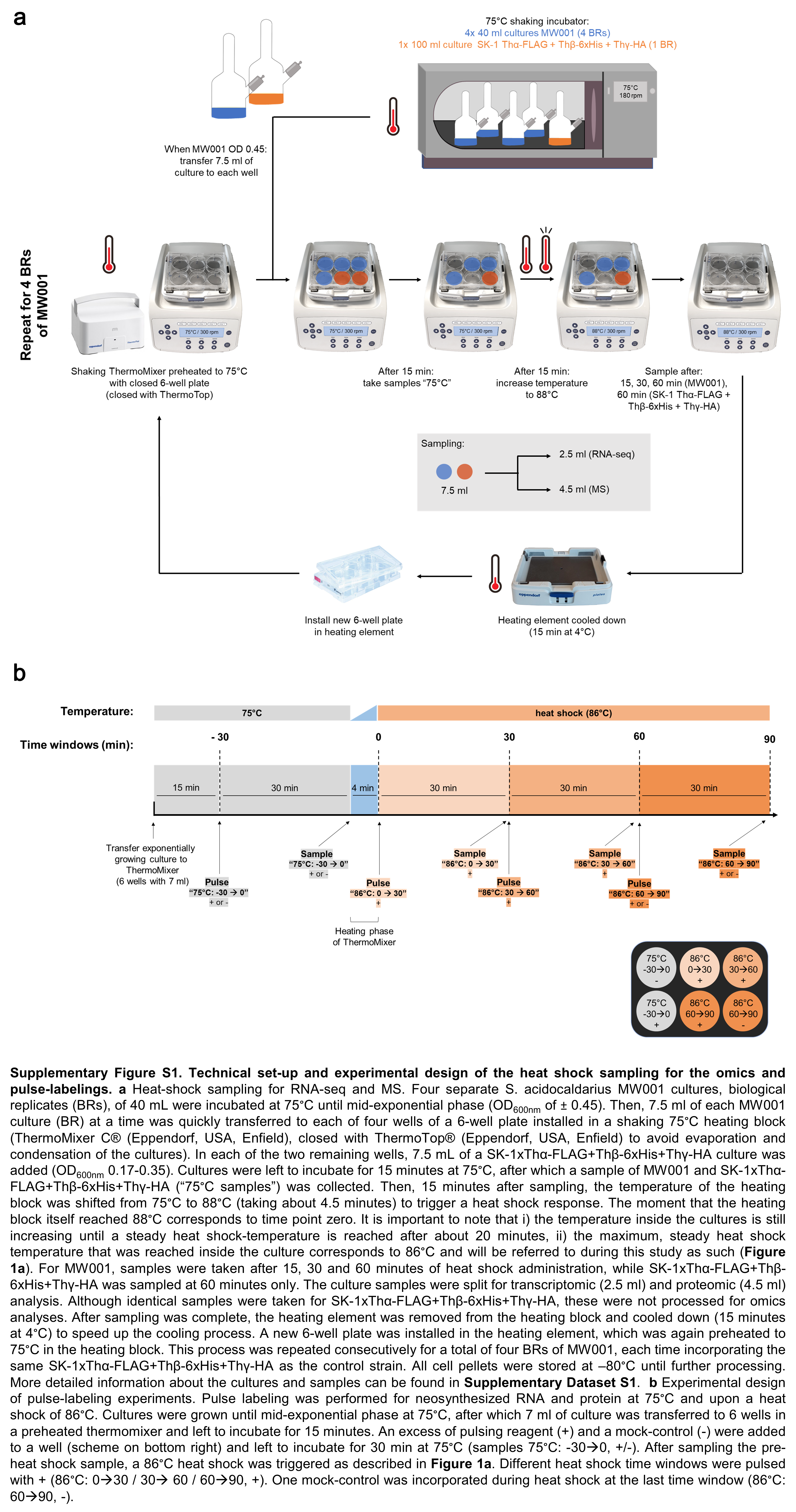

Supplement: Figure S1 — Technical set-up and experimental design of the heat shock sampling for the omics and pulse-labelings. [file mbio.03593-22-s0003.tif]

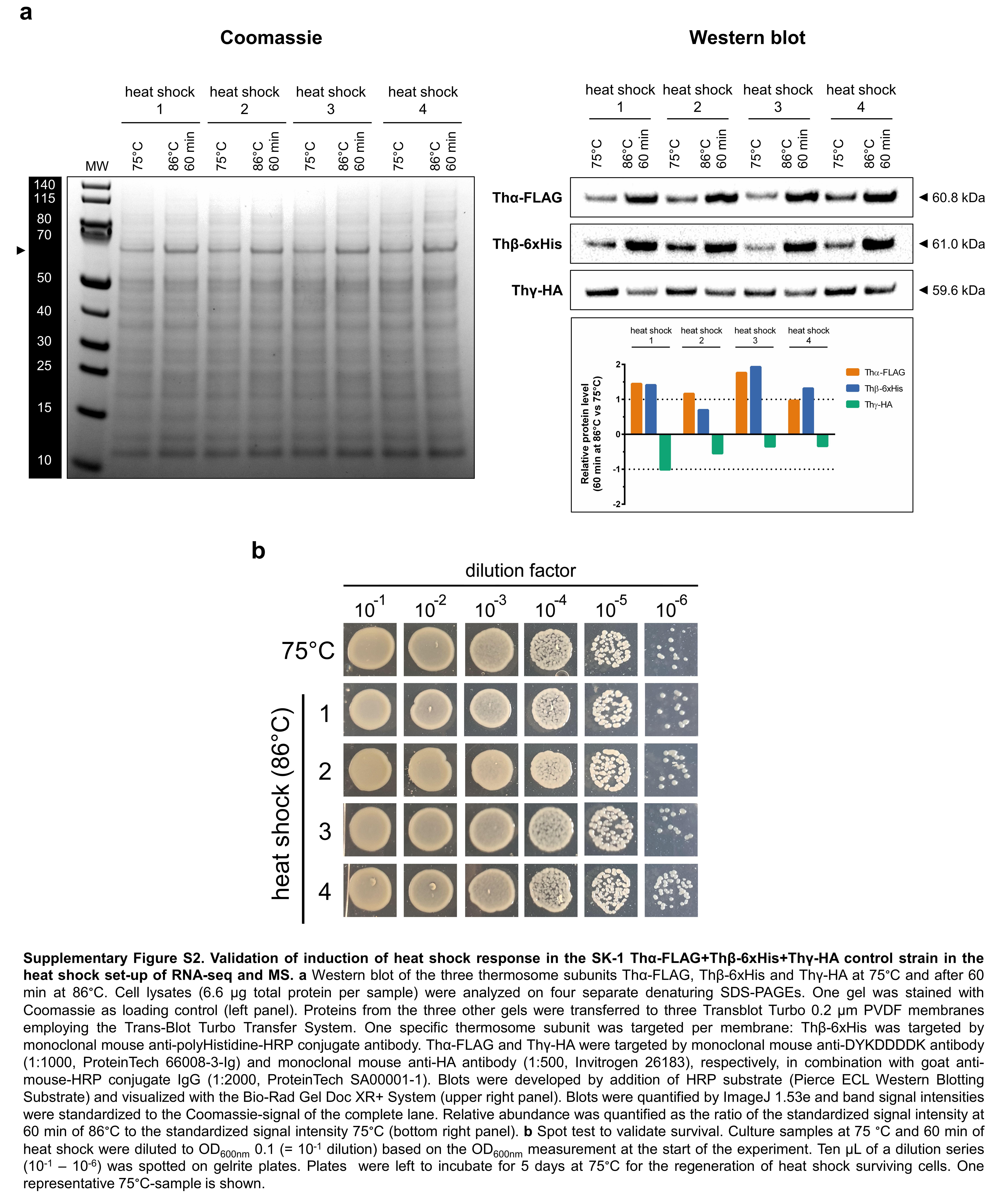

Supplement: Figure S2 — Validation of induction of heat shock response in the SK-1 Thα-FLAG+Thβ-6xHis+Thγ-HA control strain in the heat shock setup of RNA-seq and MS. [file mbio.03593-22-s0004.tif]

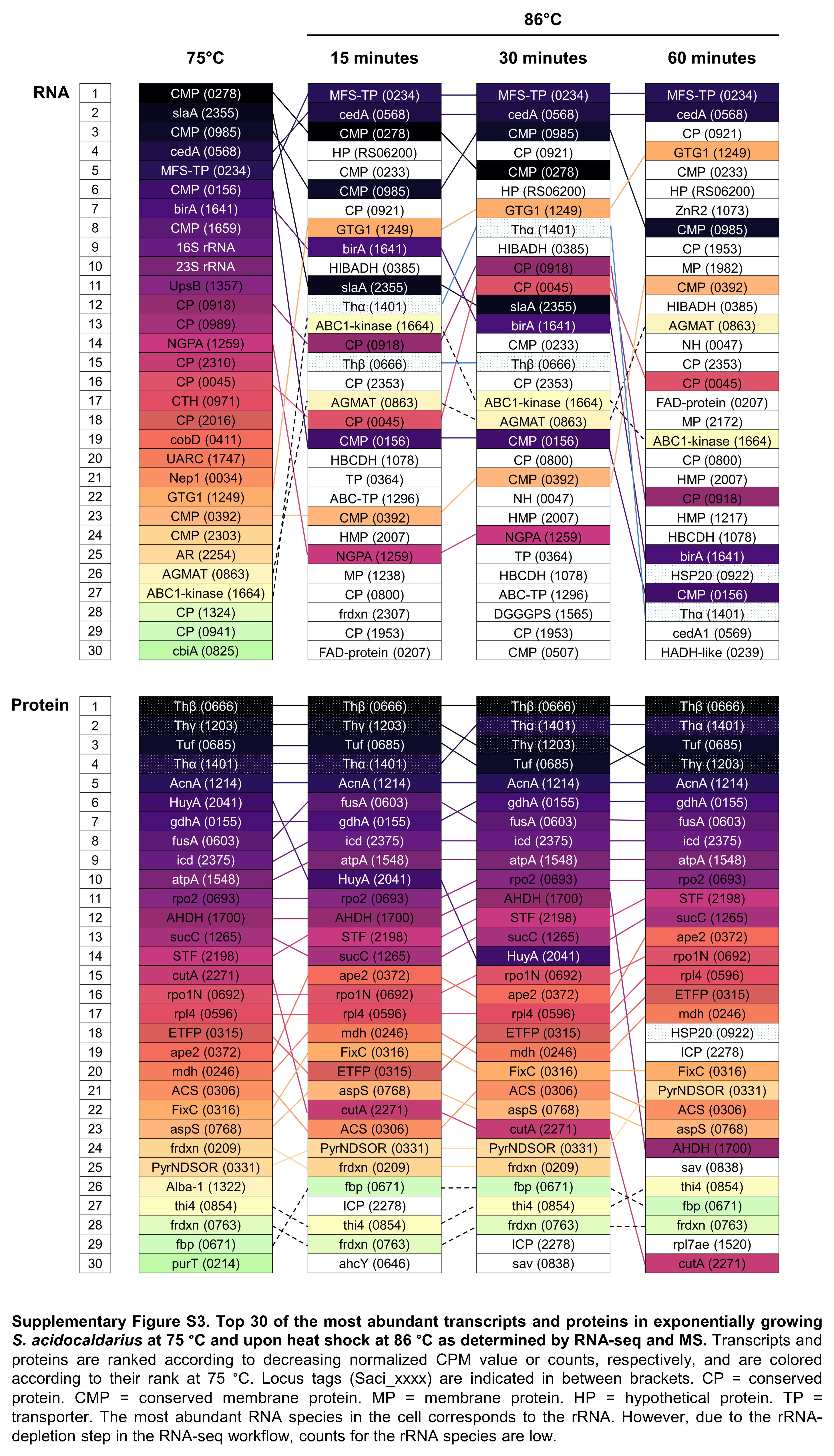

Supplement: Figure S3 — Top 30 of the most abundant transcripts and proteins in exponentially growing S. acidocaldarius at 75°C and upon heat shock. [file mbio.03593-22-s0005.tif]

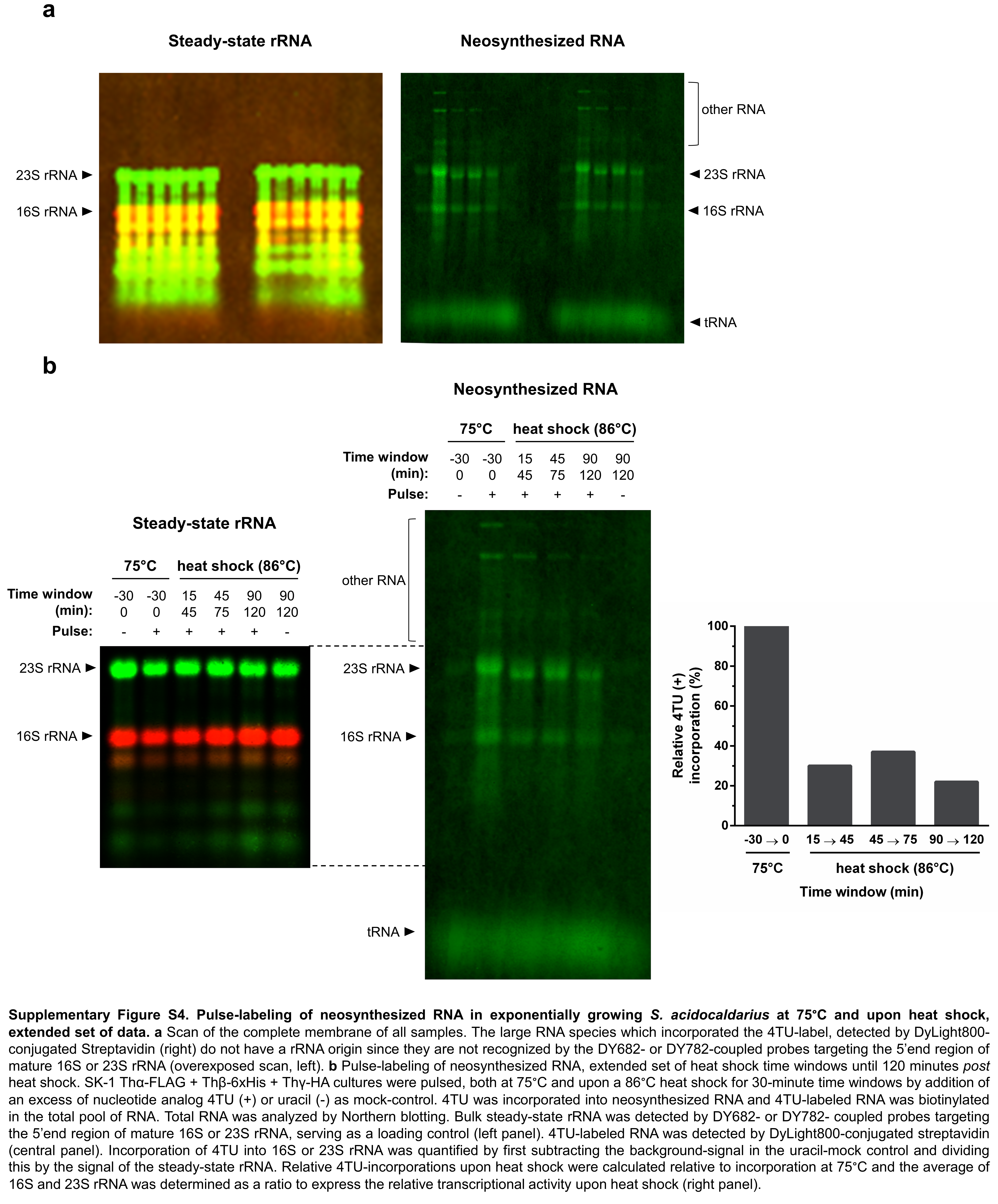

Supplement: Figure S4 — Pulse-labeling of neosynthesized RNA in exponentially growing S. acidocaldarius at 75°C and upon heat shock, extended set of data. [file mbio.03593-22-s0006.tif]

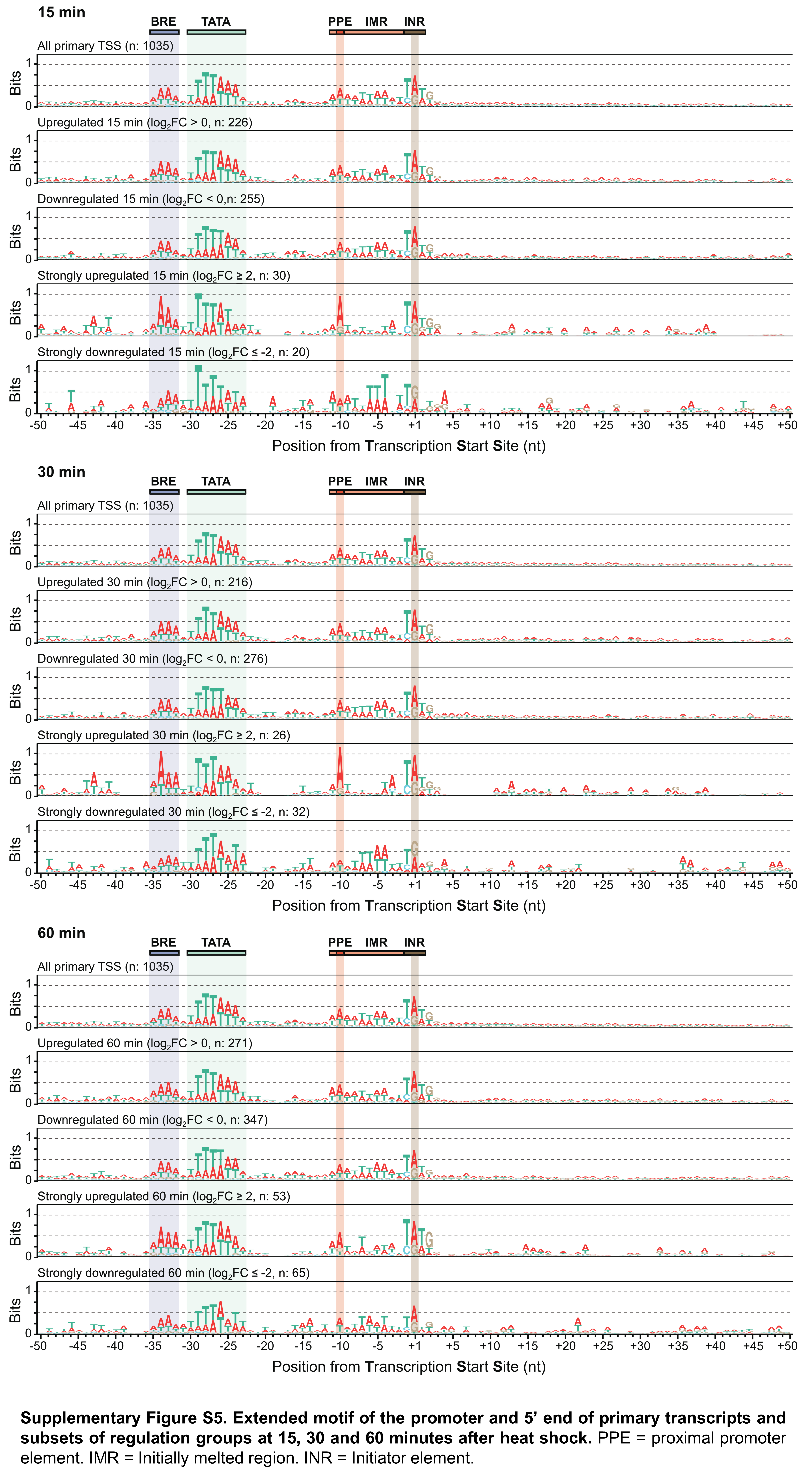

Supplement: Figure S5 — Extended motif of the promoter and 5' end of primary transcripts and subsets of regulation groups. [file mbio.03593-22-s0007.tif]
