# Supplementary material for: Transcriptional and translational dynamics underlying heat shock response in the thermophilic crenarchaeon Sulfolobus acidocaldarius
Source: mBio. 2023 Aug 29;14(5):e03593-22. doi: 10.1128/mbio.03593-22 (PMC10653856; doi:10.1128/mbio.03593-22)
Supplement: Supplemental Methods — Construction of the tagged thermosome strain, analysis of cellular viability by plating, western blotting, RNA sequencing, and TMT-labeled liquid chromatography-tandem-mass-spectrometry. [file mbio.03593-22-s0008.pdf]

Supplementary Methods. Construction of the tagged thermosome strain, analysis of cellular viability by plating, western blotting, RNA sequencing and TMT-labeled Liquid Chromatography-Tandem-Mass-Spectrometry.

Transcriptional and translational dynamics underlying heat shock response in the thermophilic Crenarchaeon *Sulfolobus acidocaldarius*

Supplementary Methods

Construction of the tagged thermosome strain

With the aim of detecting and quantifying protein levels of individual thermosome subunits, a *Sulfolobus acidocaldarius* strain was constructed harboring C-terminally tagged thermosome subunits (Thα, *Saci_1401*; Thβ, *Saci_0666*; Thγ, *Saci_1203*) with each subunit gene fused to a different tag (FLAG-, His- or HA-tag, respectively). The SK-1*x*Thα-FLAG+Thβ-6xHis+Thγ-HA strain was constructed from SK-1 (Suzuki et al., 2016) based on the pop-in/pop-out strategy as previously described (Wagner et al., 2012).

To this end, for each of the three subunit genes, genomic regions of about 1,000 base pairs (bp) up- and downstream of the intended C-terminal tag location were PCR-amplified using *S. acidocaldarius* MW001 genomic DNA as a template. Primers were designed to incorporate the desired tag. Subfragments representing the up- and downstream regions were fused in an overlap PCR approach and the resulting fragments with a length of about 2,000 bp were subsequently cloned in a BamHI/NdeI-digested pSVA431 plasmid vector, harboring *pyrEF* genes (Wagner et al., 2012),using a SLiCE cloning strategy (Zhang et al., 2012). *Escherichia coli* DH5α or MG1655 was used as a host for cloning and plasmid propagation, generating three different “pop-in” plasmids: pSVA431*x*Thα-FLAG, pSVA431*x*Thβ-6xHis and pSVA431xThγ-HA. All primers used in this work are presented in **Supplementary Methods Table 1**, all plasmids in **Supplementary Methods Table 2**.

S. acidocaldarius SK-1 electrocompetent cells were transformed by electroporation as described in (Wagner et al., 2012) with the exception that the template plasmid was not methylated. For growth on plates, 0.6 % Gelrite® (Duchefa Biochemie, Netherlands, Haarlem) was used as a solidifying agent of Brock basal salts medium supplemented with 0.2 % sucrose, 0.1 % NZ-Amine, but lacking uracil, and acidified to pH 3.0-3.5 with H_2SO_4, with addition of 3 mM $CaCl_2$ and 10 mM $MgCl_2$. After incubating during 5 days at 75°C, colonies were treated by spraying with a solution of 5 mg ml^{-1} 5-bromo-4-chloro-3-indolyl-b-D-galactopyranoside (X-gal), revealing “pop-in” integrants by blue color formation caused by LacS-activity. These colonies were further grown in liquid culture without uracil upon confirmation by PCR analysis. At mid/end-exponential phase (OD_{600} of 0.6-0.8), cells were spread on plates containing 20 µg ml^{-1} uracil and 200 µg ml^{-1} 5-fluoroorotic acid (5-FOA) and incubated for 5 days at 75°C. Candidate “pop-out” transformants were inoculated in liquid medium with uracil, and presence of the desired C-terminal tag was confirmed by PCR analysis and Sanger sequencing. This procedure was performed first for the introduction of the Thβ-6xHis tag and repeated twice for introduction of the Thα-FLAG and Thγ-HA tag, respectively, finally generating a strain harboring all three tags in the genome (SK-1*x*Thα-FLAG+Thβ-6xHis+Thγ-HA).

Supplementary Methods Table 1. DNA oligonucleotides used in this work for the construction of SK-1 (derivative) strains expressing tagged thermosome subunits. FW = forward; RV = reverse.

Name	Description	Sequence (5' → 3')
RB134	FW subfragment PCR 1,000 bp upstream Thα-FLAG = FW overlap PCR	CTCAAGCTATGCATCCAACGCGTTGGGAGCTCTCCCATATGGTGCTAGACAAAGAAGTAGTACATGCAG
RB135	RV subfragment PCR 1,000 bp upstream Thα-**FLAG**	GAGAGAGAATGTAAAATAATAAAAAATAATATACTGTATTCA**TTTATCATCATCATCTTTATAATC**CTCTAATGAAGGTGCGCCAGGAG
RB136	FW subfragment PCR 1,000 bp downstream Thα-**FLAG**	CTCCTGGCGCACCTTCATTAGAG**GATTATAAAGATGATGATGATAAA**TGAATACAGTATATTATTTTTTATTATTTTACATTCTCTCTCATTTATACATC
RB137	RV subfragment PCR 1,000 bp downstream Thα-FLAG = RV overlap PCR	GAGCCAAGTACTAGAACTGCTCAAACCTAGGTCAGGATCCGAAAAGGGATTAGTTGCGCATTG
RB037	FW subfragment PCR 1,000 bp upstream Thβ-6xHis = FW overlap PCR	CTCAAGCTATGCATCCAACGCGTTGGGAGCTCTCCCATAACAAATAGTGTATGGAATTATAGTTGATAAAGAAGTAG
RB038	RV subfragment PCR 1,000 bp upstream Thβ-**6xHis**	GATCGCACAGTATAATAAAGGTAAAAAAAGTTACTTA**GTGGTGGTGGTGGTGGTG**GTCTTCTTCTTTACCTTTTTCAGACTCTTTC
RB039	FW subfragment PCR 1,000 bp downstream Thβ-**6xHis**	GAAAGAGTCTGAAAAAGGTAAAGAAGAAGAC**CACCACCACCACCACCAC**TAAGTAACTTTTTTTACCTTTATTATACTGTGCGATC
RB040	RV subfragment PCR 1,000 bp downstream Thβ-6xHis = RV overlap PCR	GAGCCAAGTACTAGAACTGCTCAAACCTAGGTCAGGATCATCATGACATAGAATTAGGGGAAGTTATAAATAATTATG
RB138	FW subfragment PCR 1,000 bp upstream Thγ-HA = FW overlap PCR	CTCAAGCTATGCATCCAACGCGTTGGGAGCTCTCCCATATGGAGAAAGCGTAGACGAGACAACTTTAG
RB139	RV subfragment PCR 1,000 bp upstream Thγ-**HA**	CATGAGGGAAAGAAAAGAAATCCTATATTTTAACTTTTTA**TGCATAATCAGGTACATCATAAGGATA**TCCCATAGGATATTGAGGCATTTGTTG
RB140	FW subfragment PCR 1,000 bp downstream Thγ-**HA**	CAACAAATGCCTCAATATCCTATGGGA**TATCCTTATGATGTACCTGATTATGCA**TAAAAAGTTAAAATATAGGATTTCTTTTCTTTCCCTCATG
RB141	RV subfragment PCR 1,000 bp downstream Thγ-HA = RV overlap PCR	GAGCCAAGTACTAGAACTGCTCAAACCTAGGTCAGGATCCGAACCCCGGTAGTTGCATACTC
EP393	FW colony PCR cloning into pSVA431	ATGACCATGATTACGCCAAG
EP394	RV colony PCR cloning into pSVA431	TCGAACTTGCAGACAAGTTC
RB144	FW sequencing pSVA431xThα-FLAG (binding in 1,000 bp upstream)	GTGCAGTCGAGTCAGAGTTAG
RB145	RV sequencing pSVA431xThα-FLAG (binding in 1,000 bp downstream)	CTACTCATAGACGACATGAACCTAC
RB045	FW sequencing pSVA431x Thβ-6xHis (binding in 1,000 bp upstream)	GTGGCACGGAATAAATGTATATAC
RB047	RV sequencing pSVA431x Thβ-6xHis (binding in 1,000 bp downstream)	CATAATAACGCAGTCTCCTTCTAGTATAG
RB146	FW sequencing pSVA431x Thγ-HA (binding in 1,000 bp upstream)	GAACGTTATAGAGAGCCCATACA
RB147	RV sequencing pSVA431x Thγ-HA (binding in 1,000 bp downstream)	TGATCGACGTAAAGTATACCAAAGAC
RB158	FW colony PCR/sequencing "pop-in" pSVA431xThα-FLAG	CACCTTCATTAGAGGATTATAAAGATGATGATGATAAATGAATACA
RB061	FW colony PCR/sequencing "pop-in" pSVA431xThβ-6xHis	GAAGACCACCACCACCACCACCACTAAG
RB160	FW colony PCR/sequencing "pop-in" pSVA431xThγ-HA	CTATGGGATATCCTTATGATGTACCTGATTATGC
RB062	RV colony PCR/sequencing "pop-in" pSVA431(-derivatives) (binding pyrEF in pSVA431 backbone)	GATGACTACTTTAGAATATTCGAACTTGCAGACAAGTTCTATG
RB219	FW colony PCR/sequencing "pop-out": Thα-FLAG	CTCAGGTACTAAAGAGTGCTGTAGAG
RB220	RV colony PCR/sequencing "pop-out": Thα-FLAG	CTCAGTCCAGATTTTATCAACCTAGTTTTC
RB045	FW colony PCR/sequencing "pop-out": Thβ-6xHis	GTGGCACGGAATAAATGTATATAC
RB047	RV colony PCR/sequencing "pop-out": Thβ-6xHis	CATAATAACGCAGTCTCCTTCTAGTATAG
RB221	FW colony PCR/sequencing "pop-out": Thγ-HA	GAAGACGTGACCAAGGAGAACATC
RB222	RV colony PCR/sequencing "pop-out": Thγ-HA	GTGCCAAATGGTTAACGGCAATAC

Supplementary Methods Table 2. Plasmids used in this work for construction of SK-1 (derivative) strains expressing tagged thermosome subunits. Nucleotide sequences can be provided upon request.

**Name**	**Description**	**Reference**
pSVA431	Plasmid vector	(Wagner et al., 2012)
pSVA431*x*Thα-FLAG	Pop-in/pop-out plasmid for fusing thermosome α (*Saci1401*) to a C-terminal FLAG tag (DYKDDDDK).	This work
pSVA431*x*Thβ-6xHis	Pop-in/pop-out plasmid for fusing thermosome β (*Saci0666*) to a C-terminal 6xHis tag (HHHHHH).	This work
pSVA431*x*Thγ-HA	Pop-in/pop-out plasmid for fusing thermosome γ (*Saci1203*) to a C-terminal HA tag (YPYDVPDYA).	This work

Analysis of cellular viability by plating

Culture samples of SK-1*x*Thα-FLAG+Thβ-6xHis+Thγ-HA were diluted with Basic Brock medium to OD_{600nm} 0.1 (*i.e.* the 10^{-1} dilution), based on the OD_{600nm} measurement at the start of the experiment. A serial dilution series was constructed to 10^{-6} and 10 µL of each dilution were spotted on freshly prepared plates. For growth on plates, 0.6 % gelrite was used as a solidifying agent of the Brock medium with addition of 3 mM $CaCl_2$ and 10 mM $MgCl_2$. Plates were incubated for 5 days at 75°C and analyzed as previously described (Baes et al., 2020).

Western blotting

SK-1*x*Thα-FLAG+Thβ-6xHis+Thγ-HA cell pellets were resuspended in 500 µL lysis buffer (phosphate-buffered saline (PBS) pH 7.5, 1 % sodium dodecyl sulfate (SDS), 5 mM phenylmethylsulfonyl fluoride (PMSF), cOmplete™ Protease Inhibitor Cocktail (Roche, Switzerland, Basel)), lysed by ultrasonication (4 min at 70 % amplitude with 1 min pulses at 4 °C) and centrifuged for 15 minutes at 16,100 *x g*. Total protein concentrations were determined employing the Pierce™ Rapid Gold BCA Protein Assay Kit (Pierce Biotechnology, Inc., USA, Rockford).Samples were normalized to equal concentration, mixed with 4x LDS loading dye, denatured for 10 minutes at 70°C and loaded four times (6.6 µg total protein per lane) on four separate but identical denaturing sodium dodecyl sulfate (SDS) polyacrylamide gel electrophoresis (PAGE) analyses (NuPAGE™ 4-12%, Bis-Tris, 1.0 mm Mini protein gel) and ran for about 35 minutes at 200 V. One gel was stained with Coomassie as loading control. Proteins from the three other gels were transferred to three Transblot Turbo 0.2 µm polyvinylidene fluoride (PVDF) membranes employing the Trans-Blot Turbo Transfer System with default settings for a mini gel. Membranes were blocked for 1 hour with PBS + 0.1% Tween-20 + 5% skimmed milk, after which the corresponding (primary) antibodies were added in fresh blocking buffer for overnight incubation at 4°C: Thβ-6xHis was targeted by monoclonal mouse anti-polyHistidine-HRP conjugate antibody (1:1000, A7058-1VL, Sigma-Aldrich, USA, Saint Louis), Thα-FLAG was targeted by monoclonal mouse anti-DYKDDDDK antibody (1:1000, 66008-3-Ig, ProteinTech, England, Manchester) and Thγ-HA was targeted by monoclonal mouse anti-HA antibody (1:500, 26183,Invitrogen, USA, Waltham). Blots for Thα-FLAG and Thγ-HA were washed twice for 30 minutes with PBS + 0.1% Tween-20 and subjected to secondary antibody binding for 1 hour at room temperature employing goat anti-mouse-HRP conjugate IgG (1:2000, SA00001-1, ProteinTech, USA, Waltham). All three blots were subsequently washed twice for 30 minutes with PBS + 0.1% Tween-20 and 30 minutes with PBS. Blots were developed by addition of HRP substrate (Pierce ECL Western Blotting Substrate, Thermo Fisher Scientific, USA, Eugene) and visualized with the Bio-Rad Gel Doc XR+ System (Bio-Rad, USA, Hercules). Blots were quantified by ImageJ 1.53e (Schneider et al., 2012) and band signal intensities were standardized to the Coomassie signal of the complete lane.

RNA sequencing

Total RNA was extracted from MW001-stabilized cell pellets using the RNeasy Mini Kit (Qiagen, USA, Maryland) and on-column DNase treatment (Qiagen, USA, Maryland). Cell pellets were resuspended in 600 µL RLT™ lysis buffer and centrifuged for 10 minutes at 12,108 x *g* and 4°C. RNA was finally eluted from the column in 30 µL nuclease-free water. The total RNA quantity was determined with a Qubit RNA High Sensitivity Assay (Thermo Fisher Scientific, USA, Eugene) and RNA integrity was evaluated on a Bioanalyzer instrument with a RNA 6000 Nano chip (Agilent Technologies, USA, Santa Clara) (**Supplementary Dataset S1**). Ribosomal RNA depletion was established using a PAN-Archaea riboPOOL kit (siTOOLS, Germany, Planegg), followed by purification with a Zymo RNA Clean and Concentrator-5 kit (Zymo Research, USA, Irvine). Sequencing libraries were subsequently prepared with the TruSeq Stranded Total RNA Library Kit (Illumina, USA, San Diego) in combination with the RNA Unique Dual Indices (IDT for Illumina, USA, San Diego). Library enrichment PCR proceeded for 9 cycles. Library quality was verified on a Bioanalyzer instrument with a DNA High Sensitivity chip (Agilent Technologies, USA, Santa Clara) and concentrations were measured with qPCR according to 'Sequencing library quantification guide' (Illumina, USA, San Diego). Sequencing was performed on a NextSeq500 instrument (Illumina, USA, San Diego) in high output with single reads of 75 nts and 2% Phix spike-in (**Supplementary Dataset S1**).

Data were processed by first performing a quality control by FastQC (Andrews et al., 2010); quality trimming and filtering was performed by Trimmomatic (Bolger et al., 2014) (**Supplementary Dataset S1**). Sequencing reads were mapped to the *S. acidocaldarius* DSM639 genome (NC_007181.1) using STAR (Dobin et al., 2013) and read counts were produced by RSEM (Li et al., 2011). A minimum of 2182 genes out of 2351 coding genes were covered (= 92.8 %). Normalization and differential expression analysis was performed with the R-package EdgeR (Robinson et al., 2010). Genes were considered differentially expressed if the false discovery rate (FDR) was lower than 0.05 (**Supplementary Dataset S2**).

TMT-labeled Liquid Chromatography-Tandem-Mass-Spectrometry

For protein extraction, cell pellets were resuspended in 500 µL lysis buffer (PBS pH 7.5, 1% SDS, 5 mM phenylmethylsulfonyl fluoride (PMSF), cOmplete™ Protease Inhibitor Cocktail (Roche, Switzerland, Basel), lysed by ultrasonication and centrifuged for 15 minutes at 16,100 *x g*. Total protein concentrations were determined employing the Pierce™ Rapid Gold BCA Protein Assay Kit (Pierce Biotechnology, Inc., USA, Rockford) (**Supplementary Dataset S1**). Samples of 450 µL lysate were flash-frozen and stored at - 80°C. Next, 100 µL lysate samples were diluted 1:10 with 30 mM triethylammonium bicarbonate (TEAB) followed by incubation at 95°C during 5 minutes. Trypsin was added at a 1:50 ratio followed by a 5-hour incubation at 37°C and a 10-minute centrifugation at 10,000 x *g* for the collection of peptide-containing supernatants. Digested samples were stored overnight at -20°C and peptides pelleted by speedvacevaporation. Pellets were resolved in 80 µL 30 mM TEAB using water bath sonication for 2 minutes. Remaining SDS was removed employing the Pierce™ Detergent Removal Spin Columns with a 0.5-ml volume (Pierce Biotechnology, Inc., USA, Rockford) using 30 mM TEAB as equilibration buffer. Peptides were quantified with the Pierce™ Quantitative Colorimetric Peptide kit (Pierce Biotechnology, Inc., USA, Rockford). For each peptide sample, 10 µg was Tandem Mass Tag (TMT) labeled using the TMTpro 16plex Label Reagent Set (Pierce Biotechnology, Inc., USA, Rockford). Samples were multiplexed to 120 µg, dried by speedvac centrifugation and the pellets were resuspended in 100 µl of 0.5 % trifluoroacetic acid in 5 % acetonitrile. Unincorporated TMT-labels were removed on a Pierce™ C18 Spin Column (Pierce Biotechnology, Inc., USA, Rockford) and eluted in 20 µL 70 % acetonitrile. Samples were dried by speedvac centrifugation and resuspended in 0.1 % trifluoroacetic acid 3.5% acetonitrile to a final peptide concentration of 0.5 µg/µL.

One µg of peptides were directly loaded onto a reversed-phase pre-column Acclaim PepMap 100 (Thermo Fisher Scientific, USA, Eugene) and eluted in backflush mode. Peptide separation was achieved using a reversed-phase analytical column Acclaim PepMap RSLC on an Ultimate 3000 RSLN nanoHPLC system (Thermo Fisher Scientific, USA, Eugene) as described by (Ouni et al., 2022). Peptides were analyzed by mass spectrometry (MS) at an Orbitrap Fusion Lumos tribrid (Thermo Fisher Scientific, USA, Eugene) with enabled advanced peak determination (APD) and with relative quantification by MS2. Intact peptides were detected in the Orbitrap at a resolution of 120,000 with a scan range m/z from 375 to 1500 and an AGC target of $4x10^5$, maximum injection time was set to 50 ms. A data-dependent procedure of MS/MS scans was applied for the top precursor ions above a threshold ion count of 3.0×10^4 in the MS survey scan with 60 s dynamic exclusion. The total cycle time was set to 3 s. For MS2 quantification of the TMT reporter ions, MS/MS spectra were acquired in the Orbitrap at a resolution of 50,000 after HCD fragmentation at 35%, with an AGC target of 1×10^5 ions and a maximum injection time of 120 ms.

The resulting MS/MS data were processed using Sequest HT search engine within Proteome Discoverer 2.5 against the *S. acidocaldarius* reference target-decoy database obtained from NCBI (1 Jan 2022, 2,267 forward entries). Trypsin was specified as the cleavage enzyme, allowing up to two missed cleavages, four modifications per peptide, and up to three charges. Mass error was set to 10 ppm for precursor ions and 0.1 Da for fragment ions, and considered dynamic modifications were +15.99 Da for oxidized methionine and +42.011 Da for acetylation of the protein N-terminus. Fixed modifications were TMTpro (+304.207 Da) for lysine and peptide N-termini and +57.00 Da for carbamidomethyl cysteine. An overall of 1,115 proteins were detected out of the 2,267 predicted protein encoding genes (= 49.2 %). Differential protein expression analysis was performed using the DEqMS pipeline for TMT-labeled MS data (Zhu et al., 2020). To this end, protein abundance values were $\log_2$ transformed, replicate outliers removed and data normalized to have equal medians in all samples. Benjamini-Hochberg corrected p-values (Benjamini et al., 1995) were considered statistically significant at a threshold < 0.05 (**Supplementary Dataset S2**).

References

Andrews, S. FastQC: a quality control tool for high throughput sequence data (2010). Retrieved from http://www.bioinformatics.babraham.ac.uk/projects/fastqc

Baes, R., Lemmens, L., Mignon, K., Carlier, M. & Peeters, E. Defining heat shock response for the thermoacidophilic model crenarchaeon Sulfolobus acidocaldarius. *Extremophiles*. **24**, 681–692 (2020).

Benjamini, Y. & Hochberg, Y. Controlling the False Discovery Rate: A Practical and Powerful Approach to Multiple Testing. *Journal of the Royal Statistical Society: Series B (Methodological)*. **57**, 289–300 (1995).

Bolger, A. M., Lohse, M. & Usadel, B. Trimmomatic: A flexible trimmer for Illumina sequence data. *Bioinformatics*. **30**, 2114–2120 (2014).

Dobin, A. et al. STAR: Ultrafast universal RNA-seq aligner. *Bioinformatics*. **29**, 15–21 (2013).

Li, B. & Dewey, C. N. RSEM: accurate transcript quantification from RNA-Seq data with or without a reference genome. *BMC Bioinformatics*. **12**, (2011).

Ouni, E. et al. Proteome-wide and matrisome-specific atlas of the human ovary computes fertility biomarker candidates and open the way for precision oncofertility. *Matrix Biology*. **109**, 91–120 (2022).

Robinson, M. D., McCarthy, D. J. & Smyth, G. K. edgeR: A Bioconductor package for differential expression analysis of digital gene expression data. *Bioinformatics*. **26**, 139–140 (2010).

Schneider, C. A., Rasband, W. S. & Eliceiri, K. W. NIH Image to ImageJ: 25 years of image analysis. *Nature Methods*. **9**, 671–675 (2012).

Suzuki, S. & Kurosawa, N. Disruption of the gene encoding restriction endonuclease Sua I and development of a host-vector system for the thermoacidophilic archaeon Sulfolobus acidocaldarius. *Extremophiles*. **20**, 139–148 (2016).

Wagner, M. et al. Versatile genetic tool box for the crenarchaeote Sulfolobus acidocaldarius. *Front. Microbiol.* **3**, 1–12 (2012).

Zhang, Y., Werling, U. & Edelmann, W. SLiCE: a novel bacterial cell extract-based DNA cloning method. *Nucleic Acids Res.* **40**, 1–10 (2012).

Zhu, Y. et al. DEqMS: A method for accurate variance estimation in differential protein expression analysis. *Molecular and Cellular Proteomics*. **19**, 1047–1057 (2020).
