# Supplementary material for: Transcriptional and translational dynamics underlying heat shock response in the thermophilic crenarchaeon Sulfolobus acidocaldarius
Source: mBio. 2023 Aug 29;14(5):e03593-22. doi: 10.1128/mbio.03593-22 (PMC10653856; doi:10.1128/mbio.03593-22)
Supplement: Supplemental Results — Effects of heat shock on DNA replication, genome segregation and cell division, DNA repair, DNA import and motility, and biofilm formation. [file mbio.03593-22-s0009.pdf]

Supplementary Results. Effects of heat shock on DNA replication, genome segregation and cell division, DNA repair and DNA import and motility and biofilm formation.

Transcriptional and translational dynamics underlying heat shock response in the thermophilic Crenarchaeon *Sulfolobus acidocaldarius*

Supplementary Results

DNA replication, genome segregation and cell division

Unsurprisingly, considerable differential expression related to DNA replication and cell division was observed upon heat shock (**Supplementary Results Figure 1a**). Replication in *Sulfolobus* is initiated during the S-phase of the cell cycle at three independent origins (*oriC1-3*). The major regulatory step in replication initiation consists of binding of a replication initiator protein to its corresponding origin(s) (Samson et al., 2013). Our omics-results showed that the initiator proteins Cdc6-1 and Crenarchaeal-specific Whip were not displaying differential expression during heat shock (**Supplementary Results Figure 1a**). However, Cdc6-3 was transcriptionally decreased immediately after temperature shift (15-60 minutes) and Cdc6-2 protein levels were slightly decreased at 60 minutes. A previous study hypothesized that Cdc6-1 and Cdc6-3 are involved in promoting replication, given their main presence in G_1 and S phase, whereas Cdc6-2 may be a negative regulator for replication, since it is only present in G_2 (Robinson et al., 2004). It is therefore reasonable that replication initiation is not enhanced and it is even plausible that replication initiation is downregulated upon heat shock in *S. acidocaldarius*. Moreover, given the specificity of the initiator proteins (Samson et al., 2013) and the observation that Cdc6-2 is an essential transcriptional activator for upregulation of double-strand break (DSB) repair proteins (see later) (Sun et al., 2018), it is possible that firing of the different origins and activation of DSB repair is affected upon heat shock.

Cdc6-binding is followed by the recruitment of the replication elongation machinery. Upon heat shock, a transcriptional downregulation of many proteins involved, was apparent (**Supplementary Results Figure 1a**). We observed a decreased gene expression for Mcm DNA helicase (60 minutes) and a considerable transcriptional downregulation of the single-strand DNA binding protein (SSB) immediately after shift (15-60 minutes). Whereas the large subunit of replication factor C (Rfc) and proliferating cell nuclear antigen (PCNA) subunits A and B were not temperature-responsive, the small Rfc subunit and PCNA subunit C were strongly transcriptionally downregulated (15-60 minutes) and were slightly increased at the protein level (60 minutes). Transcript levels of the main replicative DNA polymerase *polB1* were transiently decreased (15-30 minutes), followed by a decrease of PolB1-binding protein *PBP-1*-encoding transcripts (60 minutes), but not *PBP-2*. In addition, *fen1* and *lig1* were transcriptionally downregulated upon heat shock, and decreased PriL and Fen1 protein levels at 60 minutes are noticeable. A persistent transcriptional downregulation was observed for *gins23* (30-60 minutes). Taken together, this might suggest that the overall process of DNA replication is slowed down upon heat shock.

Daughter chromosomes remain in close contact during the extensive G_2-phase, which is hypothesized to facilitate homologous recombination-mediated repair of the DNA when exposed to harsh environmental growth conditions (see later) (Lindås and Bernander, 2013). Although the exact mechanism is still under study, subsequent genome segregation is mediated by SegAB (Kalliomaa-Sanford et al., 2012; Lindås and Bernander, 2013) (**Supplementary Results Figure 1b**). Upon heat shock, we found thatthe SegB transcript level was unaffected, yet, its protein level was slightly increased (60 minutes). In contrast, SegA was strongly transcriptionally (15-60) and translationally (60 minutes) downregulated. Although SegA polymerization is enhanced by SegB, the decreased SegA-levels suggests that less functional segregation machinery is able to form upon heat shock.

Cell division in *S. acidocaldarius* is mediated by a set of Cdv proteins (**Supplementary Results Figure 1b**). Upon heat shock, all Cdv-encoding transcripts (*cdvA*, *cdvB*, *cdvB1*, *cdvB2*, *cdvB3* and *cdvC*) displayed a significant, continuous transcriptional decrease (15-60 minutes). In combination with the observation that growth is impaired in the *ΔcdvB1*, *ΔcdvB2* and *ΔcdvB3* mutants (Yang and Driessen, 2014; Pulschen et al., 2020), makes it unlikely that cell division upon heat shock will ensue. This is further supported by the slight transcriptional decrease in proteasomal assembly and accessory proteins

we observed upon heat shock, which are essential for proper cytokinesis (**Supplementary Results Figure 1b**).

Moreover, the genes encoding the *Sulfobus*' cell wall called S-layer, *slaA* and *slaB*, were both transcriptionally downregulated upon heat shock (**Supplementary Results Figure 1b**). Indeed, whereas the slaA-transcript was the second most abundant mRNA at 75°C, its rank was rapidly decreasing to position 64 after 60 minutes of heat shock**.** In contrast, SlaA protein was slightly increased in abundance at 60 minutes (**Supplementary Results Figure 1b**).

Thus, our study suggests that all steps of DNA replication, DNA segregation, cell division and S-layer synthesis are downregulated upon heat shock. It is therefore plausible to assume that heat shock arrests the cells in G_2 phase and induces a cell-division block, similar to stationary phase-cells (Bernander, 2007).

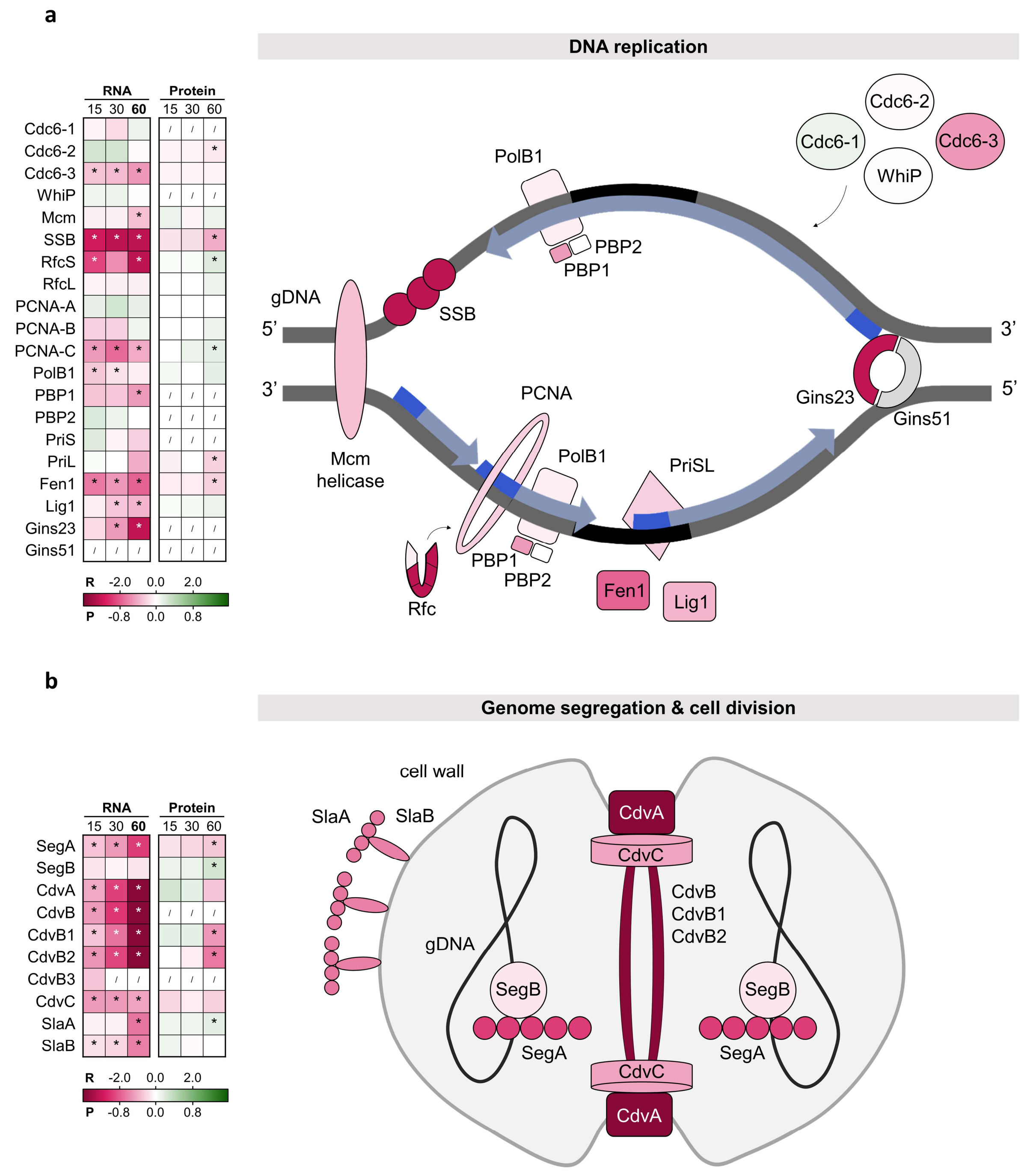

Supplementary Results Figure 1. Heat-shock responsive differential expression of the DNA replication, genome segregation and cell division machineries. The table shows the differential expression of the genes involved at the RNA (R) and protein (P) level at all time points after heat shock (15, 30, 60 minutes), color coded according to the log_2FC value in the gradient below. * = significant (FDR/adj.p-value < 0.05). / = not covered. The figure shows the proteins in their cellular context, colored according to differential expression at the RNA level after 60 minutes. a differential expression of the DNA replication machinery upon heat shock. DNA replication is initiated by binding of a replication initiator protein (Cdc6-1, Cdc6-2, Cdc6-3 and WhiP) to its corresponding origin(s) (Samson et al., 2013), followed by recruitment of Mcm DNA helicase, which unwinds the DNA helix and forms a single-stranded (ss) DNA template (Lindås and Bernander, 2013). The single-strand DNA binding protein (SSB) is involved in binding and protecting single-stranded DNA which is exposed during a variety of DNA-based processes (Napoli et al., 2005; Lindås and Bernander, 2013). The trimeric ring-shaped PCNA (Dionne et al., 2003) is loaded onto the DNA by the Rfc-complex and attaches the main replicative DNA polymerase PolB1 to the DNA template (Lindås and Bernander, 2013; Yan et al., 2017; Miyabayashi et al., 2020). PolB1 forms a heterotrimeric complex with PBP-1 and PBP-2 (Yan et al., 2017). For the lagging DNA strand, the DNA primase PriSL generates RNA primers, which are removed by the nuclease Fen1, followed by Okazaki fragment ligation by DNA ligase Lig1. Additional support of the replisome is provided by the Gins-complex (Lindås and Bernander, 2013). **b** differential expression of the genome segregation and cell division macherinery upon heat shock. Genome segregation is mediated by SegA and SegB, with SegB binding the chromosome and SegA polymerization separating the two DNA copies (Kalliomaa-Sanford et al., 2012; Lindås and Bernander, 2013). Cell division is initiated by CdvA ring-like positioning at the midcell membrane, which triggers formation of a non-contractile CdvB ring at the future division site, the latter forming a scaffold for the assembly of the CdvB1/B2 ring. Proteasomal degradation of CdvB allows for constriction of the CdvB1/B2 ring and cytokinesis (Lindås and Bernander, 2013; Risa et al., 2019; Pulschen et al., 2020). Accessory roles are played by CdvB3 and CdvC (Yang and Driessen, 2014). The cell wall of Sulfolobales, the S-layer, is composed of SlaA, anchored in the cytoplasmic membrane by SlaB (Lewis et al., 2021). Scheme adapted from Lindås and Bernander, 2013 and Lewis et al., 2021.

DNA repair and DNA import

Increasing the positively supercoiled topology of the DNA upon heat shock by the action of TopR1 is *in se* an important way to prevent DNA damage, given that this prevents formation of single-stranded (ss) DNA, which is more prone to degradation. In contrast, DNA-based processes also require opening of the double-stranded (ds) helix. Given that our results confirm the TopR1-model of DNA supercoiling upon heat shock (**Figure 4a**), our observed downregulation of the ssDNA-protecting protein SSB (**Supplementary Results Figure 1b**) and the fact that SBB enhances reverse gyrase activity in a TopR1/TopR2 mix in *Sulfolobus sp*. (Napoli et al., 2005), it is possible that SBB and TopR1 are jointly acting in the homeostatic control of DNA supercoiling and decrease of DNA replication upon heat shock.

Besides the main replicative DNA polymerase PolB1, *S. acidocaldarius* encodes three other accessory DNA polymerases (PolB2, PolB3 and PolY), of which the exact functions are not yet thoroughly understood, besides that these are mainly involved in DNA repair and DNA damage tolerance (Valenti et al., 2009; Miyabayashi et al., 2020). Upon heat shock, our results showed a decrease of *polB2* transcript levels (60 minutes) and an immediate transcriptional upregulation of *polB3* and *polY* (15-60 minutes) (**Supplementary Results Figure 2a**). A previous study showed that *S. acidocaldarius* accessory DNA polymerase single, double and triple deletion strains are more sensitive to heat stress than the wild-type strain, especially *ΔpolB3* strains (Miyabayashi et al., 2020). Furthermore, PCNA stimulates polymerase activity of PolY, which is able to bypass DNA damage, and reduces the rate of single base deletions (Wu et al., 2020). Given the differential expression of PCNA-C upon heat shock (**Supplementary Results Figure 1a**), PolY activity could be affected as well. Moreover, it was previously shown that *Sa. solfataricus* PolY is inhibited by TopR1 and directly or indirectly stimulated by SBB, thereby preventing introduction of mutations and increasing genome stability (Valenti et al., 2009). Our results thus suggest a crucial role for PolB3 and PolY in controlling DNA stability upon heat shock in *S. acidocaldarius.*

Despite the adaptation of DNA topology, spontaneous DNA damage increases at elevated temperatures *e.g.*: hydrolytic deamination and depurination, oxidation of guanines, strand breakage,… (White and Allers, 2018). The need for repair of these DNA damages is crucial in order to allow for DNA replication and maintain genome stability (Grogan, 2000). In Archaea, four universal DNA repair pathways are conserved (White and Allers, 2018), and upon heat shock, we observed considerable changes in expression level of many players involved (**Supplementary Results Figure 2a**).

In *P. furiosus*, the mismatch-specific endonuclease EndoMS (NucS) had been identified to be the key player in correction of mismatched bases, incorporated during DNA replication, by mismatch repair (MMR) (Ishino et al., 2016; White and Allers, 2018). In *S. acidocaldarius*, we found that *endoMS* is not heat shock-responsive (**Supplementary Results Figure 2a**), which is coherent with the suggestion that DNA replication is decreased upon heat shock (see above).

Structural perturbations of the DNA helix as a result of photoproduct-induced lesions are removed by nucleotide excision repair (NER) (White and Allers, 2018). The presence and exact working mechanism of NER in *Sulfolobus* is still speculative, nevertheless, putative enzymes involved were differentially expressed at the transcriptional level upon heat shock, including XPD helicase, XPB paralog *XPB1* (but not *XPB2*) and *Bax1* (**Supplementary Results Figure 2a**).

The most prevalent type of DNA damage is caused by hydrolytic depurination, deamination of cytosine, oxidation or methylation and is corrected by base excision repair (BER). Depurination is easily triggered by reactive oxygen species and the rate of deamination is increasing at higher temperatures, possibly changing base-paring propensities and increasing mutation-rate (Grasso and Tell, 2014; White and Allers, 2018). Damaged bases are detected and the glycosidic bond cleaved either by a glycosylase specific for the damaged base and AP endonuclease Endo III (Dionne and Bell, 2005; Chung et al., 2003) or endonucleases such as Endo V (White and Allers, 2018). Additional processing might occur by flap displacement, excision by the Fen1 nuclease (Grasso and Tell, 2014; White and Allers, 2018) and PCNA (Sartori and Jiricny, 2003). Upon heat shock, we observed a transcriptional downregulation

of *endo III*, *fen1* and DNA ligase and an upregulation of *endoV* (**Supplementary Results Figure 1a, Supplementary Results Figure 2a**).

The most detrimental type of DNA damage is occurring from DNA double strand breaks (DSBs), which block DNA-based processes and can induce major mutations, genome rearrangements and cell death (White and Allers, 2018). Given the absence of the non-homologous end joining pathway in archaea, the major repair pathway is homologous recombination, which is dependent on the presence of a second intact copy of the DNA (van Wolferen et al., 2015; White and Allers, 2018). Besides the use of homologous recombination in DSB repair, it is also involved in restarting DNA replication at stalled forks (White and Allers, 2018). Upon heat shock, we noted a considerable downregulation of enzymes involved in DSB end recession: gene expression of *mre11*, *rad50* (15-60 minutes) and *nurA* (15-30 minutes), and protein levels of Mre11 (30-60 minutes) and NurA (60 minutes) (**Supplementary Results Figure 2a**). Whereas HerA transcript levels were not responsive, protein levels were considerably increased (60 minutes). RadA recombinase was upregulated at the RNA level (15-60 minutes) and protein level (60 minutes). Two enzymes involved in strand exchange were transcriptionally downregulated: helicase *hel308* (15 minutes) and Holliday junction resolvase *hjr saci_1741* (15-60 minutes). However, *hjr Saci_1558* was upregulated (60 minutes). The response of *S. acidocaldarius* upon heat shock is vastly different from the response of *Sa. solfataricus* upon exposure to ionizing radiation or UV stress, where a considerable transcriptional upregulation of DSB repair genes was observed. (Fröls et al., 2007; Rolfsmeier et al., 2011). To summarize, our results suggest a decreased need for DSB end recession upon high temperature stress. However, given the upregulation of RadA (**Supplementary Results Figure 2a**) and the downregulation of SBB in the identical time-frames (**Supplementary Results Figure 1a**) and a previous finding that *Sa. solfataricus*' SSB inhibits the RadA ssDNA dependent ATPase activity (Rolfsmeier and Haseltine, 2010), it is plausible to assume that RadA has a key role in DSB repair upon heat shock.

Most of the time a second copy of the *S. acidocaldarius* chromosome is present in the cell, since most cells are in G_2-phase (Lindås and Bernander, 2013) and our results suggest that DNA replication and cell division is slowed down upon heat shock. However, it has been previously described for *S. acidocaldarius* that, upon exposure to UV irradiation, the UV-inducible (ups) pili and the Crenarchaeal system for exchange of DNA (Ced) are transcriptionally upregulated and are involved in species-specific aggregation and chromosomal DNA import, respectively (Fröls et al., 2007). This imported DNA copy is serving as a template for the repair of DSBs by homologous recombination (Ajon et al., 2011; van Wolferen et al., 2013, 2015, 2016). In our study, and in contrast to UV-stress (Fröls et al., 2007), we observed no massive transcriptional upregulation of the ups-genes upon heat shock (**Supplementary Results Figure 2b**). However, a large transcriptional upregulation was observed for *cedA*, *cedA1*, *cedA2* and the HerA-homolog immediately after heat shock (15-60 minutes), associated with a downregulation of *cedB* (**Supplementary Results Figure 2b**). Whereas cedA was already amongst the most abundant transcripts in the cell at 75°C, it is the second most abundant transcript upon heat shock.

A transcriptional upregulation was observed for most of the DNA-processing enzymes encoded downstream of *ups*, which are thought to be involved in subsequent DNA processing (van Wolferen et al., 2015), including *endo III* nuclease, a glycosyltransferase and the helicase Hel112, and a translational upregulation of the ParB-like nuclease (**Supplementary Results Figure 2b**).

Upon UV-stress, it has been shown previously that the transcription initiation factor TFB3 is upregulated at 45 minutes and is serving as an essential activator for *ups*- and *ced* transcription at 90 minutes (Schult et al., 2018). However, read counts for TFB3 were low in our RNA-Seq and MS analyses, so it remains to be assessed whether TFB3 is required in the observed heat shock-responsive upregulation of *ced* gene expression. However, given that the upregulation of *ced* was already observed very rapidly at 15 minutes of heat shock (**Supplementary Results Figure 2b**), it seems unlikely that this is dependent on prior upregulation of TFB3 (given that its abundance is low at 75°C). Thus, our results suggest that, besides the 2nd 'own' genomic copy, there is an additional need for intact DNA copies to enhance the

repair the DSB induced by heat shock in *Sulfolobus*, but possibly haploid Crenarchaea in general. Alternatively, this might pose a way to increase genetic diversity upon temperature stress.

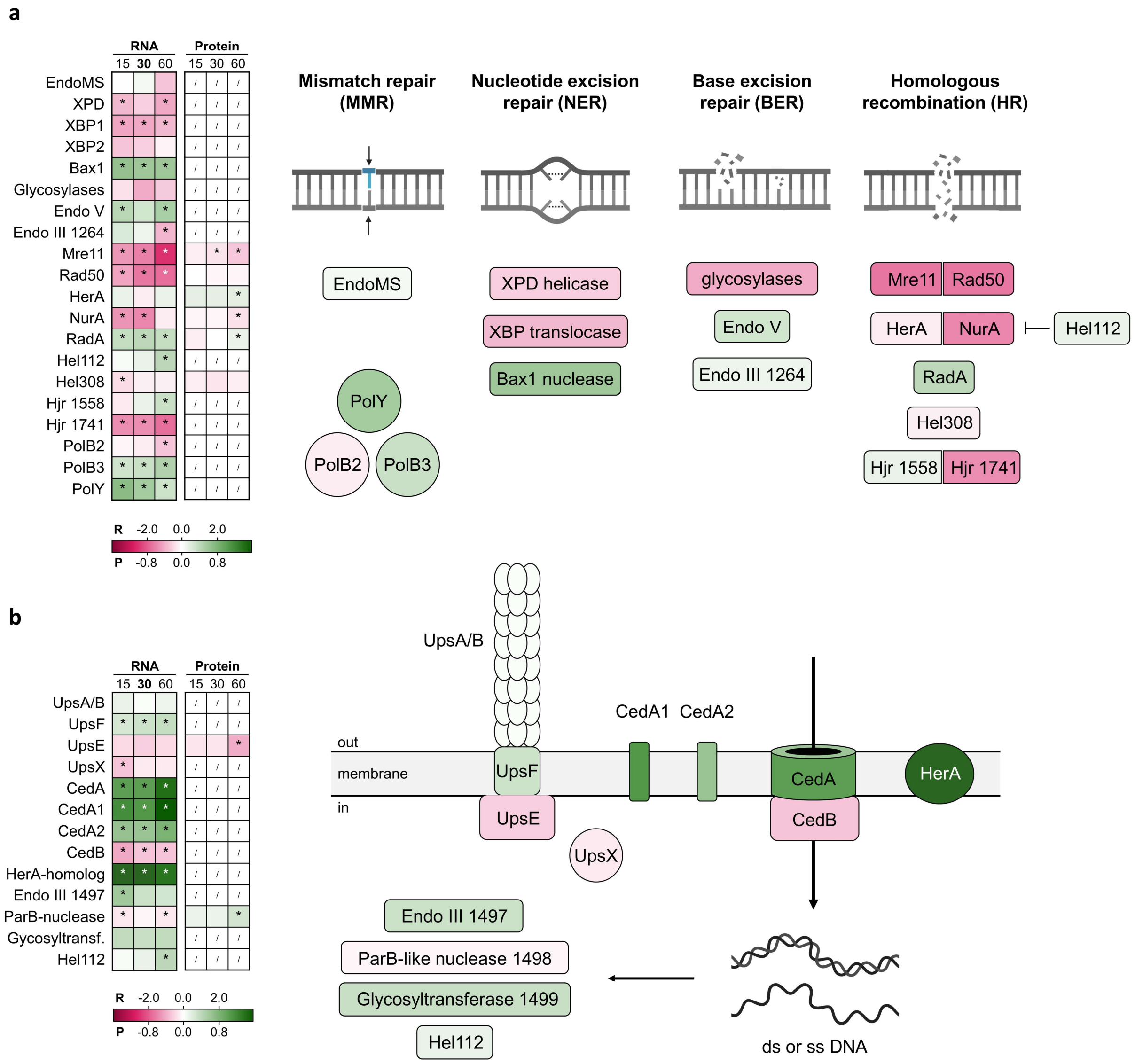

Supplementary Results Figure 2. Differential expression of the DNA repair mechanisms and the Crenarchaeal system for exchange of DNA upon heat shock. The table shows the differential expression of the genes involved at the RNA (R) and protein (P) level at all time points after heat shock (15, 30, 60 minutes), color coded according to the $\log_2$FC value in the gradient below. * = significant (FDR/adj.p-value < 0.05). / = not covered. The figure shows the proteins in their cellular context, colored according to differential expression at the RNA level after 30 minutes. **a** Differential expression of the universally conserved DNA repair mechanisms upon heat shock. The three accessory DNA polymerases (PolB2, PolB3 and PolY) are involved in DNA repair (Valenti et al., 2009; Miyabayashi et al., 2020). Mismatched nucleotides generated during DNA replication are detected and repaired by EndoMS in MMR (Ishino et al., 2016; White and Allers, 2018). NER is involved in the repair of helix-destabilizing lesions induced by photoproducts. The XPD helicase is essential for DNA unwinding, and the complex consisting of XPB dsDNA translocase and Bax1 endonuclease assists in repair (Richards et al., 2008; White and Allers, 2018). Hydrolytic depurination of the DNA, deamination of cytosine, oxidation or methylation is corrected by BER. In the canonical BER pathway, a glycosylase specific for the damage base detects the lesion and cleaves the glycosidic bond (Dionne and Bell, 2005) and AP endonuclease Endo III (Chung et al., 2003) detects an abasic nucleotide and cleaves the DNA backbone, allowing a DNA polymerase to initiate DNA repair synthesis and DNA ligase to ligate the nick. Additional processing might occur by flap displacement and excision by Fen1 nuclease (Grasso and Tell, 2014; White and Allers, 2018). In the alternative BER pathway, endonucleases such as endoV are involved in nicking the DNA next to the DNA lesion instead of a glycosylase (White and Allers, 2018). In Sulfolobus, repair of DNA double stand breaks (DSBs) is mainly established by homologous recombination and requires the presence

of a second intact DNA copy (White and Allers, 2018). Repair is initiated by DSB end recession by the 5' to 3' exonuclease complex Mre11-Rad50 and helicase/nuclease complex HerA-NurA, yielding 3' ssDNA ends which form nucleoprotein filaments with RadA recombinase, after displacement of SBB. The nucleoprotein filament engages in an homology search with the intact DNA copy and catalyzes strand exchange and the formatting of a displacement (D-) loop. The 3'end of the invading template strand in the D-loop is either unwound by the helicase Hel308 or the D-loop may capture the second end of the DNA DSB, forming a four-way Holliday junction, which is then resolved by Holliday junction resolvases (Hjr) and possibly inducing cross-over (White and Allers, 2018). **b** Differential expression of the ced DNA import system upon heat shock. The ups pili, built from UpsA and UspB pilin subunits and anchored in the membrane by UspF and ATPase UpsE (van Wolferen et al., 2013), are involved in species-specific cellular aggregation (Ajon et al., 2011). The Ced-system consists of four transmembrane proteins (CedA, CedA1, CedA2, CedB) and a putative Ced-associated ATPase (HerA-homolog). CedA and CedB are required for chromosomal DNA import, either in a ssDNA or dsDNA form (Van Wolferen et al., 2016). An ups-neighboring gene cluster, consisting of the Endo III, a ParB-like nucleases, a glycosyltransferase and helicase Hel112, is involved in subsequent DNA processing (van Wolferen et al., 2015). Note that it is still unknown how DNA export is established (Van Wolferen et al., 2016).

Motility and biofilm formation

Heat shock also induces considerable effects on the expression of the major cellular appendages, the adhesive type IV pili (aap-pili) and the archaellum, and their regulators (**Supplementary Results Figure 3**). Whereas the aap-pili are indispensable for surface adhesion and biofilm formation (Koerdt et al., 2010; Henche et al., 2012), rotary motion of the archaellum drives cell motility (Nuno de Sousa Machado et al., 2022).

The pilus consists of AapA and AapB pilins (Henche et al., 2012), which showed a different trend of expression upon heat shock: *aapA* expression was strongly upregulated and *aapB* was strongly downregulated (**Supplementary Results Figure 3**). The motor complex anchor protein *aapE* was downregulated (15-60 minutes) and *aapF* was strongly upregulated (15-60 minutes). However, both motor proteins were decreased at the translational level during persistent heat shock. Upon temperature shift, transcript levels of the archaellin filament (*arlB*) remained unchanged, however, we observed a transcriptional downregulation of the *arlX* scaffold (30 minutes) and upregulation of the *arlG* and *arlJ* motor (**Supplementary Results Figure 3**).

Regulation of the process of biofilm formation, motility, and the switch between a free-living, motile, and sessile life style, is highly complex and dependent on many regulators and their phosphorylation status (Orell et al., 2013; Haurat et al., 2017; Bischof et al., 2019). Our results demonstrate that all known regulators display differential expression upon heat shock (**Supplementary Results Figure 3**). Besides its architectural role as a NAP (**Figure 4b**), the Lrs14-type proteins AbfR1 and Saci_1223 are thought to be key players in regulating these processes: AbfR1 activates motility, while repressing biofilm formation and Saci_1223 activates biofilm formation (Orell et al., 2013). Upon heat shock, strikingly, both AbfR1 and Saci_1223 were strongly upregulated at the RNA and protein level (**Supplementary Results Figure 3**). In addition, RNA levels of the archaellum activators *arnR* and *arnR1* activators were increased, while the RNA level of the ArnS archaellum activator was decreased. In addition, we observed a fast, persistent transcriptional downregulation of the archaellum repressors ArnA and ArnB, kinase ArnD and phosphatase PP2A upon heat shock. In contrast, the kinase *arnC* was only transiently downregulated.

Under optimal growth conditions in rich, liquid medium, archaellum expression is low (Haurat et al., 2017). However, previous studies have shown that motility is enhanced when *S. acidocaldarius* cells are exposed to high temperatures (Lewus and Ford, 1999) or upon nutrient starvation (Haurat et al., 2017). The ArnS activator plays an important role in starvation-induced motility (Haurat et al., 2017). In contrast, heat shock-induced motility is most likely not dependent on ArnS, given the lack of transcriptional upregulation observed for this regulator (**Supplementary Results Figure 3**).

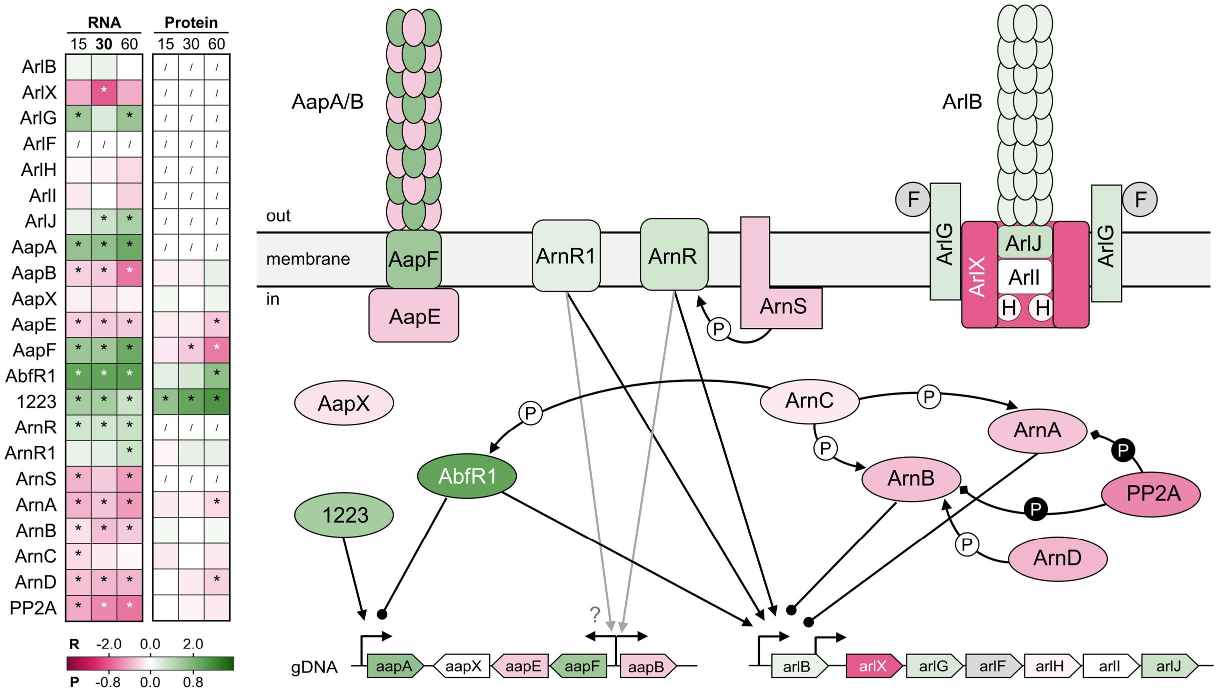

Supplementary Results Figure 3. Differential expression of the of the aap-pilus and archaellum upon heat shock. The table shows the differential expression of the genes involved at the RNA (R) and protein (P) level at all time points after heat shock (15, 30, 60 minutes), color coded according to the $\log_2$FC value in the gradient below. * = significant (FDR/adj.p-value < 0.05). / = not covered. The figure shows the proteins in their cellular context, colored according to DE at the RNA level after 30 minutes. The aap-pilus is involved in biofilm formation and consists of AapA and AapB pilins, anchored in the membrane by the AapF and AapE motor proteins (Henche et al., 2012). AapX, a putative iron-sulfur oxidoreductase, is encoded in the same gene cluster (Henche et al., 2012). The archaellum is the motility structure. All seven structural components of the archaellum (ArlB, ArlX, ArlF-J) are encoded in an operon of which transcription is controlled by two promotors, upstream of arlB and upstream of arlX (Lassak et al., 2012). Regulation of both cellular processes is intertwined. The Lrs14-type AbfR1 is the major archaellum transcriptional activator and biofilm repressor (Orell et al., 2013). The membrane-bound one-component transcription factors ArnR and its paralog ArnR1 are activators for archaellum transcriptional activation upon, e.g. nutrient starvation (Lassak et al., 2013), and are involved in regulating aap-expression. The transcriptional regulators ArnA and ArnB negatively regulate archaellum-transcription and are phosphorylated and activated by the kinases ArnC (phosphorylating ArnA and ArnB) and ArnD (phosphorylating ArnB only) and can be dephosphorylated by the PP2A Ser/Thr phosphatase (Reimann et al., 2012, 2013). Arrow = positive regulation (activation), dot = negative regulation (repression). P on white background = phosphorylation, P on black background = dephosporylation. The scheme is adapted from Bischof et al., 2019 and Nuno de Sousa Machado et al., 2022.

References

Ajon, M., Fröls, S., van Wolferen, M., Stoecker, K., Teichmann, D., Driessen, A.J.M., Grogan, D.W., Albers, S.V., and Schleper, C. (2011). UV-inducible DNA exchange in hyperthermophilic archaea mediated by type IV pili. Mol. Microbiol. *82*, 807–817. https://doi.org/10.1111/j.1365-2958.2011.07861.x.

Bernander, R. (2007). The cell cycle of Sulfolobus. Mol. Microbiol. *66*, 557–562. https://doi.org/10.1111/j.1365-2958.2007.05917.x.

Bischof, L.F., Haurat, M.F., and Albers, S. V. (2019). Two membrane-bound transcription factors regulate expression of various type-IV-pili surface structures in Sulfolobus acidocaldarius. PeerJ *2019*, 1–18. https://doi.org/10.7717/peerj.6459.

Chung, J.H., Im, E.K., Park, H.Y., Kwon, J.H., Lee, S., Oh, J., Hwang, K.C., Lee, J.H., and Jang, Y. (2003). A novel uracil-DNA glycosylase family related to the helix-hairpin-helix DNA glycosylase superfamily. Nucleic Acids Res. *31*, 2045–2055. https://doi.org/10.1093/nar/gkg319.

Dionne, I., and Bell, S.D. (2005). Characterization of an archaeal family 4 uracil DNA glycosylase and its interaction with PCNA and chromatin proteins. Biochem. J. *387*, 859–863. https://doi.org/10.1042/BJ20041661.

Dionne, I., Nookala, R.K., Jackson, S.P., Doherty, A.J., and Bell, S.D. (2003). A heterotrimeric PCNA in the hyperthermophilic archaeon Sulfolobus solfataricus. Mol. Cell *11*, 275–282. https://doi.org/10.1016/S1097-2765(02)00824-9.

Fröls, S., Gordon, P.M.K., Panlilio, M.A., Duggin, I.G., Bell, S.D., Sensen, C.W., and Schleper, C. (2007). Response of the hyperthermophilic archaeon Sulfolobus solfataricus to UV damage. J. Bacteriol. *189*, 8708–8718. https://doi.org/10.1128/JB.01016-07.

Grasso, S., and Tell, G. (2014). Base excision repair in Archaea: Back to the future in DNA repair. DNA Repair (Amst). *21*, 148–157. https://doi.org/10.1016/j.dnarep.2014.05.006.

Grogan, D.W. (2000). The question of DNA repair in hyperthermophilic archaea. Trends Microbiol. *8*, 180–185. https://doi.org/10.1016/S0966-842X(00)01729-7.

Haurat, M.F., Figueiredo, A.S., Hoffmann, L., Li, L., Herr, K., J. Wilson, A., Beeby, M., Schaber, J., and Albers, S.V. (2017). ArnS, a kinase involved in starvation-induced archaellum expression. Mol. Microbiol. *103*, 181–194. https://doi.org/10.1111/mmi.13550.

Henche, A.L., Ghosh, A., Yu, X., Jeske, T., Egelman, E., and Albers, S.V. (2012). Structure and function of the adhesive type IV pilus of Sulfolobus acidocaldarius. Environ. Microbiol. *14*, 3188–3202. https://doi.org/10.1111/j.1462-2920.2012.02898.x.

Ishino, S., Nishi, Y., Oda, S., Uemori, T., Sagara, T., Takatsu, N., Yamagami, T., Shirai, T., and Ishino, Y. (2016). Identification of a mismatch-specific endonuclease in hyperthermophilic Archaea. Nucleic Acids Res. *44*, 2977–2986. https://doi.org/10.1093/nar/gkw153.

Kalliomaa-Sanford, A.K., Rodriguez-Castañeda, F.A., McLeod, B.N., Latorre-Roselló, V., Smith, J.H., Reimann, J., Albers, S. V., and Barillà, D. (2012). Chromosome segregation in Archaea mediated by a hybrid DNA partition machine. PNAS *109*, 3754–3759. https://doi.org/10.1073/pnas.1113384109.

Koerdt, A., Gödeke, J., Berger, J., Thormann, K.M., and Albers, S.V. (2010). Crenarchaeal Biofilm Formation under Extreme Conditions. PLoS One *5*. https://doi.org/10.1371/journal.pone.0014104.

Lassak, K., Neiner, T., Ghosh, A., Klingl, A., Wirth, R., and Albers, S.V. (2012). Molecular analysis of the crenarchaeal flagellum. Mol. Microbiol. *83*, 110–124. https://doi.org/10.1111/j.1365-2958.2011.07916.x.

Lassak, K., Peeters, E., Wróbel, S., and Albers, S.V. (2013). The one-component system ArnR: A membrane-bound activator of the crenarchaeal archaellum. Mol. Microbiol. *88*, 125–139. https://doi.org/10.1111/mmi.12173.

Lewis, A.M., Recalde, A., Bräsen, C., Counts, J.A., Nussbaum, P., Bost, J., Schocke, L., Shen, L., Willard, D.J., Quax, T.E.F., et al. (2021). The biology of thermoacidophilic archaea from the order Sulfolobales. FEMS Microbiol. Rev. *45*, 1–60. https://doi.org/10.1093/femsre/fuaa063.

Lewus, P., and Ford, R.M. (1999). Temperature-sensitive motility of Sulfolobus acidocaldarius influences

population distribution in extreme environments. J. Bacteriol. *181*, 4020–4025. .

Lindås, A.C., and Bernander, R. (2013). The cell cycle of archaea. Nat. Rev. Microbiol. *11*, 627–638. https://doi.org/10.1038/nrmicro3077.

Miyabayashi, H., Jain, R., Suzuki, S., Grogan, D.W., and Kurosawa, N. (2020). PolB1 Is Sufficient for DNA Replication and Repair Under Normal Growth Conditions in the Extremely Thermophilic Crenarchaeon Sulfolobus acidocaldarius. Front. Microbiol. *11*. https://doi.org/10.3389/fmicb.2020.613375.

Napoli, A., Valenti, A., Salerno, V., Nadal, M., Garnier, F., Rossi, M., and Ciaramella, M. (2005). Functional interaction of reverse gyrase with single-strand binding protein of the archaeon Sulfolobus. Nucleic Acids Res. *33*, 564–576. https://doi.org/10.1093/nar/gki202.

Nuno de Sousa Machado, J., Albers, S.-V., and Daum, B. (2022). Towards Elucidating the Rotary Mechanism of the Archaellum Machinery. Front. Microbiol. *13*. https://doi.org/10.3389/fmicb.2022.848597.

Orell, A., Peeters, E., Vassen, V., Jachlewski, S., Schalles, S., Siebers, B., and Albers, S.-V. (2013). Lrs14 transcriptional regulators influence biofilm formation and cell motility of Crenarchaea. ISME J. *7*, 1886–1898. https://doi.org/10.1038/ismej.2013.68.

Pulschen, A.A., Mutavchiev, D.R., Culley, S., Sebastian, K.N., Roubinet, J., Roubinet, M., Risa, G.T., van Wolferen, M., Roubinet, C., Schmidt, U., et al. (2020). Live Imaging of a Hyperthermophilic Archaeon Reveals Distinct Roles for Two ESCRT-III Homologs in Ensuring a Robust and Symmetric Division. Curr. Biol. *30*, 2852-2859.e4. https://doi.org/10.1016/j.cub.2020.05.021.

Reimann, J., Lassak, K., Khadouma, S., Ettema, T.J.G., Yang, N., Driessen, A.J.M., Klingl, A., and Albers, S.V. (2012). Regulation of archaella expression by the FHA and von Willebrand domain-containing proteins ArnA and ArnB in Sulfolobus acidocaldarius. Mol. Microbiol. *86*, 24–36. https://doi.org/10.1111/j.1365-2958.2012.08186.x.

Reimann, J., Esser, D., Orell, A., Amman, F., Pham, T.K., Noirel, J., Lindås, A.C., Bernander, R., Wright, P.C., Siebers, B., et al. (2013). Archaeal signal transduction: Impact of protein phosphatase deletions on cell size, motility, and energy metabolism in sulfolobus acidocaldarius. Mol. Cell. Proteomics *12*, 3908–3923. https://doi.org/10.1074/mcp.M113.027375.

Richards, J.D., Cubeddu, L., Roberts, J., Liu, H., and White, M.F. (2008). The Archaeal XPB Protein is a ssDNA-Dependent ATPase with a Novel Partner. J. Mol. Biol. *376*, 634–644. https://doi.org/10.1016/j.jmb.2007.12.019.

Risa, G.T., Hurtig, F., Bray, S., Hafner, A.E., Harker-Kirschneck, L., Faull, P., Davis, C., Papatziamou, D., Mutavchiev, D.R., Fan, C., et al. (2019). Proteasome-mediated protein degradation resets the cell division cycle and triggers ESCRT-III-mediated cytokinesis in an archaeon. BioRxiv 1–12. .

Robinson, N.P., Dionne, I., Lundgren, M., Marsh, V.L., Bernander, R., and Bell, S.D. (2004). Identification of Two Origins of Replication in the Single Chromosome of the Archaeon Sulfolobus solfataricus. Cell *116*, 25–38. https://doi.org/10.1016/S0092-8674(03)01034-1.

Rolfsmeier, M.L., and Haseltine, C.A. (2010). The Single-Stranded DNA Binding Protein of Sulfolobus solfataricus Acts in the Presynaptic Step of Homologous Recombination. J. Mol. Biol. *397*, 31–45. https://doi.org/10.1016/j.jmb.2010.01.004.

Rolfsmeier, M.L., Laughery, M.F., and Haseltine, C.A. (2011). Repair of DNA double-strand breaks induced by ionizing radiation damage correlates with upregulation of homologous recombination genes in sulfolobus solfataricus. J. Mol. Biol. *414*, 485–498. https://doi.org/10.1016/j.jmb.2011.10.020.

Samson, R.Y., Xu, Y., Gadelha, C., Stone, T.A., Faqiri, J.N., Li, D., Qin, N., Pu, F., Liang, Y.X., She, Q., et al. (2013). Specificity and Function of Archaeal DNA Replication Initiator Proteins. Cell Rep. *3*, 485–496. https://doi.org/10.1016/j.celrep.2013.01.002.

Sartori, A.A., and Jiricny, J. (2003). Enzymology of Base Excision Repair in the Hyperthermophilic Archaeon Pyrobaculum aerophilum. J. Biol. Chem. *278*, 24563–24576. https://doi.org/10.1074/jbc.M302397200.

Schult, F., Le, T.N., Albersmeier, A., Rauch, B., Blumenkamp, P., Van Der Does, C., Goesmann, A., Kalinowski, J., Albers, S.V., and Siebers, B. (2018). Effect of UV irradiation on Sulfolobus acidocaldarius and involvement of the general transcription factor TFB3 in the early UV response. Nucleic Acids Res. *46*, 7179–7192. https://doi.org/10.1093/nar/gky527.

Sun, M., Feng, X., Liu, Z., Han, W., Liang, Y.X., and She, Q. (2018). An Orc1/Cdc6 ortholog functions as a key regulator in the DNA damage response in Archaea. Nucleic Acids Res. *46*, 6697–6711. https://doi.org/10.1093/nar/gky487.

Valenti, A., Perugino, G., Nohmi, T., Rossi, M., and Ciaramella, M. (2009). Inhibition of translesion DNA polymerase by archaeal reverse gyrase. Nucleic Acids Res. *37*, 4287–4295. https://doi.org/10.1093/nar/gkp386.

White, M.F., and Allers, T. (2018). DNA repair in the archaea-an emerging picture. FEMS Microbiol. Rev. *42*, 514–526. https://doi.org/10.1093/femsre/fuy020.

van Wolferen, M., Ajon, M., Driessen, A.J.M., and Albers, S.V. (2013). Molecular analysis of the UV-inducible pili operon from Sulfolobus acidocaldarius. Microbiologyopen *2*, 928–937. https://doi.org/10.1002/mbo3.128.

van Wolferen, M., Ma, X., and Albers, S.V. (2015). DNA processing proteins involved in the UV-induced stress response of sulfolobales. J. Bacteriol. *197*, 2941–2951. https://doi.org/10.1128/JB.00344-15.

Van Wolferen, M., Wagner, A., Van Der Does, C., and Albers, S.V. (2016). The archaeal Ced system imports DNA. Proc. Natl. Acad. Sci. U. S. A. *113*, 2496–2501. https://doi.org/10.1073/pnas.1513740113.

Wu, Y., Jaremko, W.J., Wilson, R.C., and Pata, J.D. (2020). Heterotrimeric PCNA increases the activity and fidelity of Dbh, a Y-family translesion DNA polymerase prone to creating single-base deletion mutations. DNA Repair (Amst). https://doi.org/10.1016/j.dnarep.2020.102967.

Yan, J., Beattie, T.R., Rojas, A.L., Schermerhorn, K., Gristwood, T., Trinidad, J.C., Albers, S. V., Roversi, P., Gardner, A.F., Abrescia, N.G.A., et al. (2017). Identification and characterization of a heterotrimeric archaeal DNA polymerase holoenzyme. Nat. Commun. *8*, 1–15. https://doi.org/10.1038/ncomms15075.

Yang, N., and Driessen, A.J.M. (2014). Deletion of cdvB paralogous genes of Sulfolobus acidocaldarius impairs cell division. Extremophiles *18*, 331–339. https://doi.org/10.1007/s00792-013-0618-5.
