# Supplementary material for: Transcriptional and translational dynamics underlying heat shock response in the thermophilic crenarchaeon Sulfolobus acidocaldarius
Source: mBio. 2023 Aug 29;14(5):e03593-22. doi: 10.1128/mbio.03593-22 (PMC10653856; doi:10.1128/mbio.03593-22)
Supplement: Table S1 — Microbial strains used in this study. [file mbio.03593-22-s0010.pdf]

Supplementary Table S1. Microbial strains used in this study.

**Name**	**Description**	**Reference**
S. acidocaldarius DSM639	*S. acidocaldarius* wild-type strain	DSMZ
S. acidocaldarius MW001	Uracil-auxotrophic (*ΔpyrEF*) strain	(Wagner et al., 2012)
S. acidocaldarius SK-1	Uracil-auxotrophic (*ΔpyrEF*) and SuaI restriction system-deficient (*ΔsuaI*) strain	(Suzuki et al., 2016)
S. acidocaldarius SK-1*x*Thβ-6xHis	SK-1 expressing thermosome β (Saci_0666) fused to a C-terminal 6xHis-tag (HHHHHH)	This study
S. acidocaldarius SK-1*x*Thα-FLAG+ Thβ-6xHis	SK-1 expressing thermosome α (Saci_1401) fused to a C-terminal FLAG-tag (DYKDDDDK) and thermosome β (Saci_0666) fused to a C-terminal 6xHis-tag (HHHHHH)	This study
S. acidocaldarius SK-1*x*Thα-FLAG+ Thβ-6xHis+Thγ-HA	SK-1 expressing thermosome α (Saci_1401) fused to a C-terminal FLAG-tag (DYKDDDDK), thermosome β (Saci_0666) fused to a C-terminal 6xHis-tag (HHHHHH) and thermosome γ (Saci_1203) fused to a C-terminal HA-tag (YPYDVPDYA)	This study
E. coli DH5α	Strain for plasmid cloning and propagation	Gibco
E. coli MG1655	Strain for plasmid cloning and propagation	Gibco

Suzuki, S. & Kurosawa, N. Disruption of the gene encoding restriction endonuclease Sua I and development of a host-vector system for the thermoacidophilic archaeon Sulfolobus acidocaldarius. *Extremophiles*. **20**, 139–148 (2016).

Wagner, M. et al. Versatile genetic tool box for the crenarchaeote Sulfolobus acidocaldarius. *Front. Microbiol.* **3**, 1–12 (2012).
